# Supplementary material for: Dynamics of CLIMP-63 S-acylation control ER morphology
Source: Nat Commun. 2023 Jan 17;14:264. doi: 10.1038/s41467-023-35921-6 (PMC9844198; doi:10.1038/s41467-023-35921-6)
Supplement: Supplementary file 1 — Supplementary Information [file 41467_2023_35921_MOESM1_ESM.pdf]

Supplementary Information

Supplementary Methods

Fluorescent-based Quantitative Acylation Detection

Non-acylated fraction. CLIMP-63-HA was immunoprecipitated as previously described. After 3 washes in IP buffer, beads were directly eluted in the elution buffer (100 mM HEPES, 1 mM EDTA, 2.5% SDS). NH_2OH (Sigma) was added or not at the final concentration of 1 M at pH 7.4. Beads were incubated 30 min at 50°C, vortexed and spun down. The supernatant was retrieved and NH_2OH was removed using Amicon centrifugal filter units 10kDa MWCO (Millipore, US), including 3 washes with alkylation buffer (100 mM HEPES, 1 mM EDTA, 1% SDS). The concentrated sample was then incubated with 200 µM Oregon-Green 488-iodoacetamide (Thermo, US) for 3 h in a thermal-shaker at 500 rpm at RT in the dark. Finally, samples were processed for SDS-PAGE and Western blot. Gels are first visualized by Typhoon Trio (GE Healthcare, US) or Fusion FX (Vilber Lourmat, CH) before transfer for western blot.

qPCR Primers

**Table -1 QPCR primers used in this study**	
ATF6	forward : GGTGCTCTGGAACAGGGCTC
	reverse : GTCTCCCCTTCTGCGGATGG
BIP	forward : GCTGCCCAACTGGCTGGCAA
	reverse : GCCGACGCAGGAGTAGGTGG
CALNEXIN	forward : CCGGAAGCCCGAGGATTGGG
	Reverse : CTTCATCTGGAATCTTAGCAGGGGC
IRE1	forward : GCTGTCAGCAAGAGGACAGGC
	reverse : GGCTGCCATCATTAGGATCTGGG
PERK	forward : GGTGAAGCCAATGACAGTAGCTGG
	reverse: CGTCCCAGGCATTGAATTGGCTC
CLIMP-63	forward : CTTTCTCGGGCTGGTGCGTCC
	reverse : TGGCTTGCAAAGACTGCACCTTCT
ZDHHC6	forward : GGGCTTTGTCCCTCTGGGG
	reverse : GCCAAGCATACAAGGCACCACG
spliced XBP1_f	forward : TGCCCTGGTTGCTGAAGAG
spliced XBP1_r	reverse: TGCACCTGCTGCGGACT
unspliced XBP1_f	forward : CAGCACTCAGACTACGTGCACC
unspliced XBP1_r	reverse: GGTGACAACTGGGCCTGC

Computational Modeling

The core kinetic structure of the model is based on the approach employed in previous palmitoylation models[1,2] namely the application of tQSSA (total quasi-steady-state assumption) to all enzymatic reactions, in this case all palmitoylation and depalmitoylation reactions with various ZDHHC or APT enzymes. This assumption is built upon approximating the dynamics of enzyme-substrate complexes. tQSSA is desirable in this cases due to its agreement with mass-action rate expressions, its handling of both low and high extremes of enzyme concentration, as well as its consideration of competition between substrates[3,4]. All other reaction steps are modelled with elementary mass-action rate laws.
This approximation has already been successfully used in models of multiple phosphorylation/dephosphorylation cycles[3,4] which bear a certain similarity to the palmitoylation system studied here.

In order to automatically generate an ODE (Ordinary Differential Equation) system, rule-based modeling with RuleBender was used [5].

In order to solve the ODE system the Sundials [6] interface of DifferentialEquations.jl [7] was used within Julia 0.6

As in previous modeling efforts concerning modeling palmitoylated proteins[1], no previous information concerning the various parameters of the model were known, leading to the use of an optimization algorithm to try and estimate model parameters. To cope with difficulties in finding a suitable parameter space CMA-ES (Covariance Matrix Adaptation – Evolutionary Strategy) was used [8,9]. The fitness function was calculated by integrating the area contained between the simulated model output and the piecewise linear curve built by connecting the experimental data points. The CMA-ES algorithm was run with a population size of 1000 until convergence, defined as less than 0.1 % change in the fitness function. The 100 parameter sets with the best fitness function were then selected for validation and generating predictions

All data used for calibration and validation as well as the model output are available in Figure 4 and Supplementary Fig. 4.

Global Sensitivity Analysis was performed (the list of parameters is shown in Supplementary Fig. 5c) [10] on the fitness function used to calibrate the model. Both first

order and total effect Sobol sensitivity indices of each parameter were calculated. Samples for global sensitivity analysis were generated with a combined latin hypercube/full factorial design with 16 levels in the parameter space defined by the 100 select parameter sets, 65’000 samples were generated, such that the change in the Sobol indices converged with increasing sample size, defined as a change of less than 5 %. This global sensitivity analysis served two purposes: firstly, to assess which parameters are most important for the accurate calibration for the model. Secondly, these results were used to corroborate the conclusions and predictions subsequently made with the model.

The following assumptions were made in order to define the model structure and build the ensuing system of ODEs:

- Transport of CLIMP-63 from the ER to the PM may have a time delay. To take this into account, the model includes a 3rd transportation compartment, that brings CLIMP63 from the ER to the PM. No reactions are implemented in this compartment; its purpose is merely to simulate the time taken for transportation to the ER. Transport rates to and from this compartment are modeled with mass-action kinetics. Rather than assume certain values for these parameters, they are estimated along with all other parameters with CMA-ES.
- ZDHHC6 is located in the ER and ZDHHC(2+5) in the PM. This is based on experimental knowledge as to the localization of these enzymes and their activity on CLIMP-63.
- Transport to the PM is unidirectional, CLIMP-63 is not transported back to the ER. This is generally the case for proteins with a role at the PM, they are synthesized in the ER and transported to their site of activity. *N.B.* Models with bi-directional transport were also tested, but did not behave in a significantly different manner than those with uni-directional transport.
- There is a corresponding APT in the ER and PM that depalmitoylate CLIMP-63. Pulse-chase experiments performed show that CLIMP-63 depalmitoylates under control conditions and ZDHHC6 siRNA. Given that transport is uni-directional, it is possible that there are 2 APTs, one in each compartment, that depalmitoylate CLIMP-63.
- Protein degradation occurs in both the ER and PM, albeit at different and independent rates since the degradation routes are likely to differ.

- A higher ordered (H) form of CLIMP-63 exists in the ER. Our H form shares most of the same properties as elementary units (Es – trimeric units of CLIMP-63): it is palmitoylated by ZDHHC6, depalmitoylated by the same APT, and can also be degraded. The rates and kinetic constants for the palmitoylation, depalmitoylation and degradation are however different for E and H forms. Furthermore, both hetero- and homo-Hs can be formed, meaning that the CLIMP63 molecules forming the the H complexes are independently palmitoylated and depalmitoylated.
- It is assumed that Hs are locked to the ER, once formed Hs (dimers of E) have reached a mature, functional state, and are no longer transported away from the ER. *N.B.* Models incorporating transport Hs were also tested, but did not behave in a significantly different fashion. We did not differentiate the structure of H complexes (dimers of Es in cis-parallel or trans-antiparallel across the ER lumen). Currently there is no data to support a specific conformation.
- Hs and Es both compete for the same APT and ZDHHC in the ER, ie. these enzymes are active on both the elementary units and dimeric forms, albeit with different Michaelis constants. This is implemented in the rate expressions built using tQSSA.
- The same dimerization (H-formation) constant was used for non-palmitoylated and palmitoylated monomers.

Persistent Homology Background

One view of persistent homology is that of a mathematical machine that turns a *simplicial complex* into a *vector space*. A simplicial complex is a kind of combinatorically defined topological space that can be thought of as a higher-dimensional generalization of a graph; instead of just having vertices and edges, and rules relating how edges connect vertices, simplicial complexes in addition have triangles (and rules relating each one to three edges), tetrahedra (and rules relating each one to four triangles), and so forth. In a simplicial complex, a vertex is also called a 0-*simplex*, an edge a 1-simplex, a triangle a 2-simplex, etc. Such a transition from *topology* to *algebra* can be a useful way to extract information about the underlying space, or, in applications such as this, about the data underlying that space.

A collection of *p*-simplices is called a *p-chain*. It is formally written as a sum of simplices. Of fundamental importance in algebraic topology is the *boundary* of a p-chain c, which intuitively is the $(p-1)$-chain ∂c consisting of the $(p-1)$-simplices that are faces of all the p-simplices that make up c. For example, in the annex figure a below, the red 1-chain is the boundary of the 2-chain consisting of the three grey triangles (2-simplices) which it encloses. A *p-cycle* is a p-chain without a boundary; in the figure the 1-cycles are the green, red and blue chains, while the yellow one is not

a

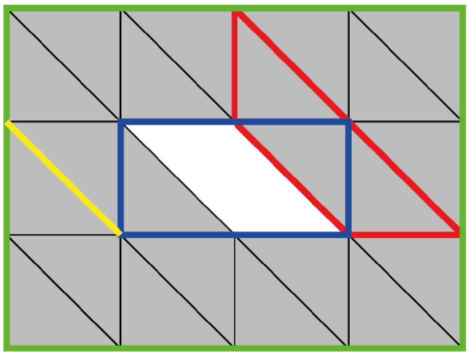

Annex figure a. A simplicial complex K with 20 0-simplices, 38 1-simplices and 22 2-simplices and some highlighted 1-chains. The yellow 1-chain consists of a single 1-simplex, and is neither a cycle nor a boundary. The red 1-chain has trivial boundary, and is therefore a cycle. It is not a representative of any non-trivial homology class, for it is the boundary of 2-chain consisting of the three 2- simplices it encloses. The green and the blue 1-chains are cycles that represent the same homology class (intuitively the 2-dimensional hole in the middle) because the green one can be obtained from the blue one by adding to the latter the boundary of the 2-simplices in between them. $H_1(K)$ is thus 1-dimensional due to the central hole.

a cycle because its boundary consists of its two endpoint vertices. Observe that every p-chain that is the boundary of a $(p + 1)$-chain is a p-cycle, but the converse is not true.

Annex figure **a** illustrates how the geometrically intuitive notion of a *hole* can be formulated precisely as “the cycles that are not boundaries”, for example the green and the blue cycles, but not the red. This is exactly what *homology* captures. The *degree-p* homology of a simplicial complex K, written $H_p(K)$ is the vector space whose elements are collections of p-cycles that differ by a boundary. In other words, $Hp(K)$ is the equivalence class of p-cycles modulo those that are boundaries of $(p + 1)$-chains. Figure **a** illustrates the idea for 1-cycles. For a higher-degree example, the reader may imagine a (hollow) sphere built up from triangles and think about what a non-bounding 2-cycle is in that case.

Which domain-specific properties homology captures, i.e. “the meaning of holes”, is of course wholly dependent on how the simplicial complex is built from observed data. We now describe one such construction whose close relative is used in the actual ER analysis. Let X be a finite set of points in some Euclidean space $\mathbb{R}^d$. For some fixed radius $r > 0$, we consider the set of all (closed) balls of radius r centred at the points of X. Č$_r$ (K) is a simplicial complex whose 0-simplices are the points in X, and whose p-simplices $(p > 0)$ are given by the $(p + 1)$-fold intersections of the aforementioned balls.

Annex figure **b** shows the idea. If we let r vary from zero to infinity, we obtain a sequence of simplicial complexes *Č*$_r$ (X) with the property that Č$_r$ $(X) \subseteq$ Č$_s$ (X) whenever $r \leq s$. *Persistent homology* is a way of tracking the creation (“birth”) and destruction

b

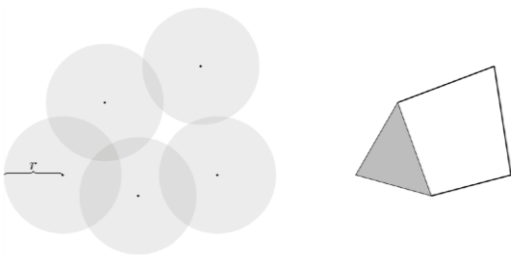

Annex Figure b. Left: A point cloud X with five points, and balls (disks) of some radius r centred at them. Right: The Čech complex Čr (X). The vertices are drawn at the coordinates of the points of X for visualization purposes only.

(“death”) of homology classes across such a filtration.

We summarize persistent homology of a filtration in a *persistence diagram*, which is simply a (multi)set of points above the diagonal in the plane. Each point at coordinates (b, d) signifies a homology class born at filtration radius b and dying at radius d. Points far off the diagonal, i.e. points with d much greater than b, thus represent "robust" or in some sense "big" holes (loops, cavities, etc.). Points near the diagonal, i.e. point with d only marginally bigger than b, represent "noise" or "small" holes. These points, and their distribution, can be thought of as a fingerprint of the hole structure of the simplicial complex.

It is this property of persistent homology that we use to quantitatively distinguish between the cavity sizes (degree-2 homology) or the membrane arrangement (degree-1 homology) in two ER reconstructions.

As input we take the vertices of the reconstructed ER as a point cloud in $\mathbb{R}^3$. A filtration is constructed in a way that is very similar to the Čech filtration described above, but with a slight variation: we do not consider intersections of the balls themselves, but rather the balls intersected with the *Voronoi cells*[11] of the point cloud. Given a fixed collection of points X in Euclidean space, the Voronoi cell of a point $x \in X$ is the part of space that is at least as close to x as it is to any other point in X. This *Alpha complex filtration*[12] has a similar geometric interpretation to the Čech complex, but crucially has far fewer simplices and is thus computationally much more tractable since it clearly disallows some simplices forms by vastly overlapping balls.

Computations for persistent homology were done GUDHI software2.0.0: @book{gudhi
, title = "{GUDHI} User and Reference Manual"
, author = "{The GUDHI Project}"
, publisher = "{GUDHI Editorial Board}"
, year = 2015
ad-hoc scripts and code were used for analysis rendering and visualisation of persistence diagrams.

Supplementary Figures

Supplementary Figure 1

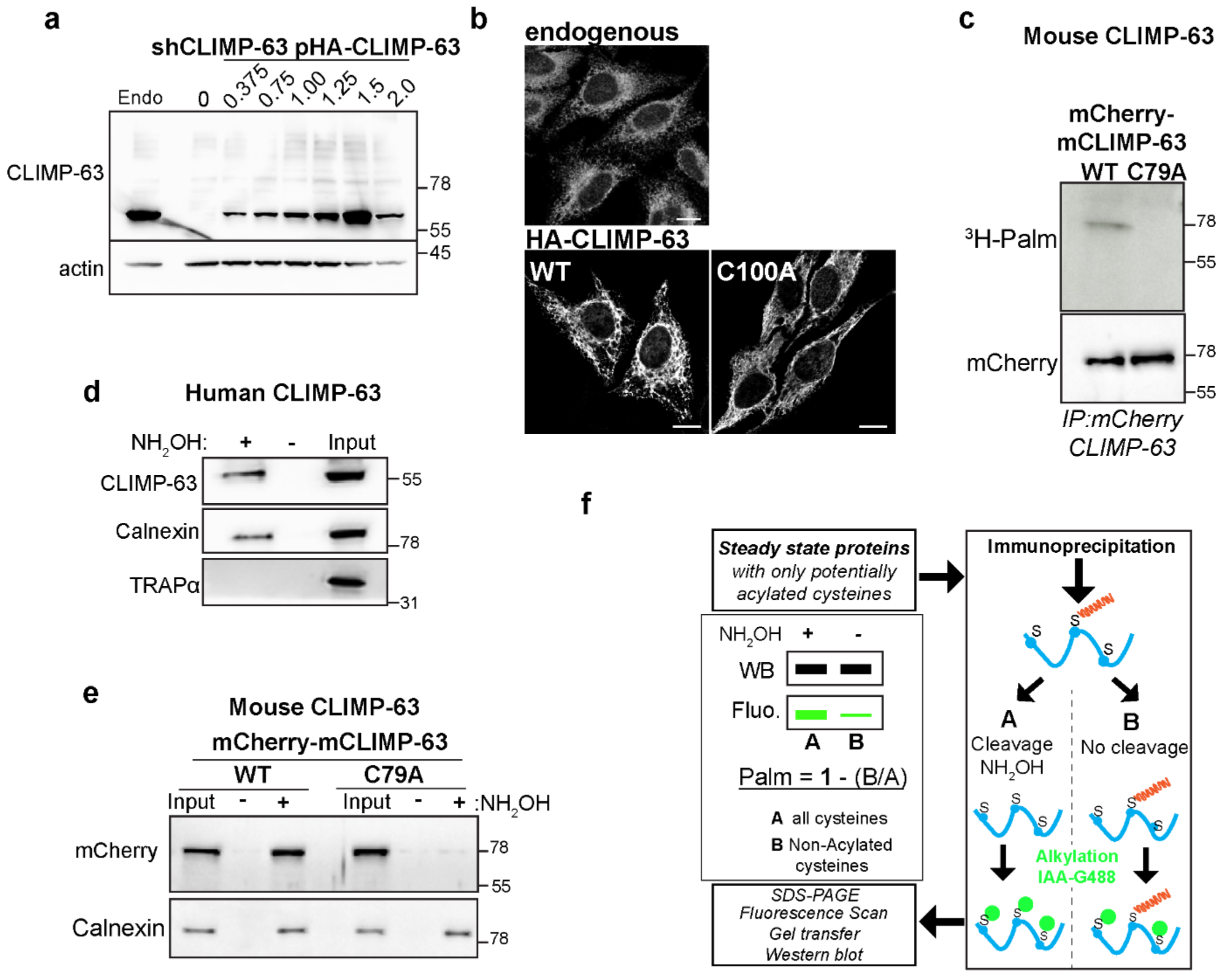

Supplementary Figure 1. CLIMP-63 S-acylation. a. Comparative analysis of the level of endogenous CLIMP-63 in HeLa cells (Endo) with HA-CLIMP-63 expressed in shCLIMP-63 stable HeLa cells transfected with the indicated DNA amount (µg/well of 6 well plate). **b.** Immunofluorescence of endogenous CLIMP-63 in HeLa cells or HA-CLIMP-63 (WT or C100A mutant) in shCLIMP-63 transfected for 48 h. Scale bar: 10 µm. **c**. ^{3}H-palmitate labelling of HeLa cells expressing murine mcherry-mCLIMP-63 (WT or C79A mutant). Western blot and autoradiography show ^{3}H-palmitate in mcherry-mCLIMP-63 immunoprecipitation fractions. **d. e.** Acyl-RAC assay for capturing acylated protein species from HeLa post-nuclear supernatants following specific cleavage with hydroxylamine (NH2OH). The input corresponds to the same amount of protein extract used in each condition for the pull-downs. In **e**. cells were transfected as in **c. f.** Schematic description of the method used in Fig. 1c. Following cell harvest the target protein is immunoprecipitated. Acylated cysteines are cleaved, or not, by NH2OH. Free cysteines in both samples are then labelled by a second alkylation step using iodoacteamide-oregon-green-488 (IAA-OG488). Proteins are separated by SDS-PAGE and IAA-OG488 incorporation is analysed by fluorescence imaging. The same gel is subsequently processed for Western blot analysis to control for the presence of target protein in the IP fractions. Two conditions are used to quantify the population of acylated cysteines. In sample-A the cleaved cysteines indicate the total amount of cysteines whereas B corresponds to the non-acylated cysteines. Thus, acylation is 100% minus the ratio of non-acylated to total cysteine.

Supplementary Figure 2

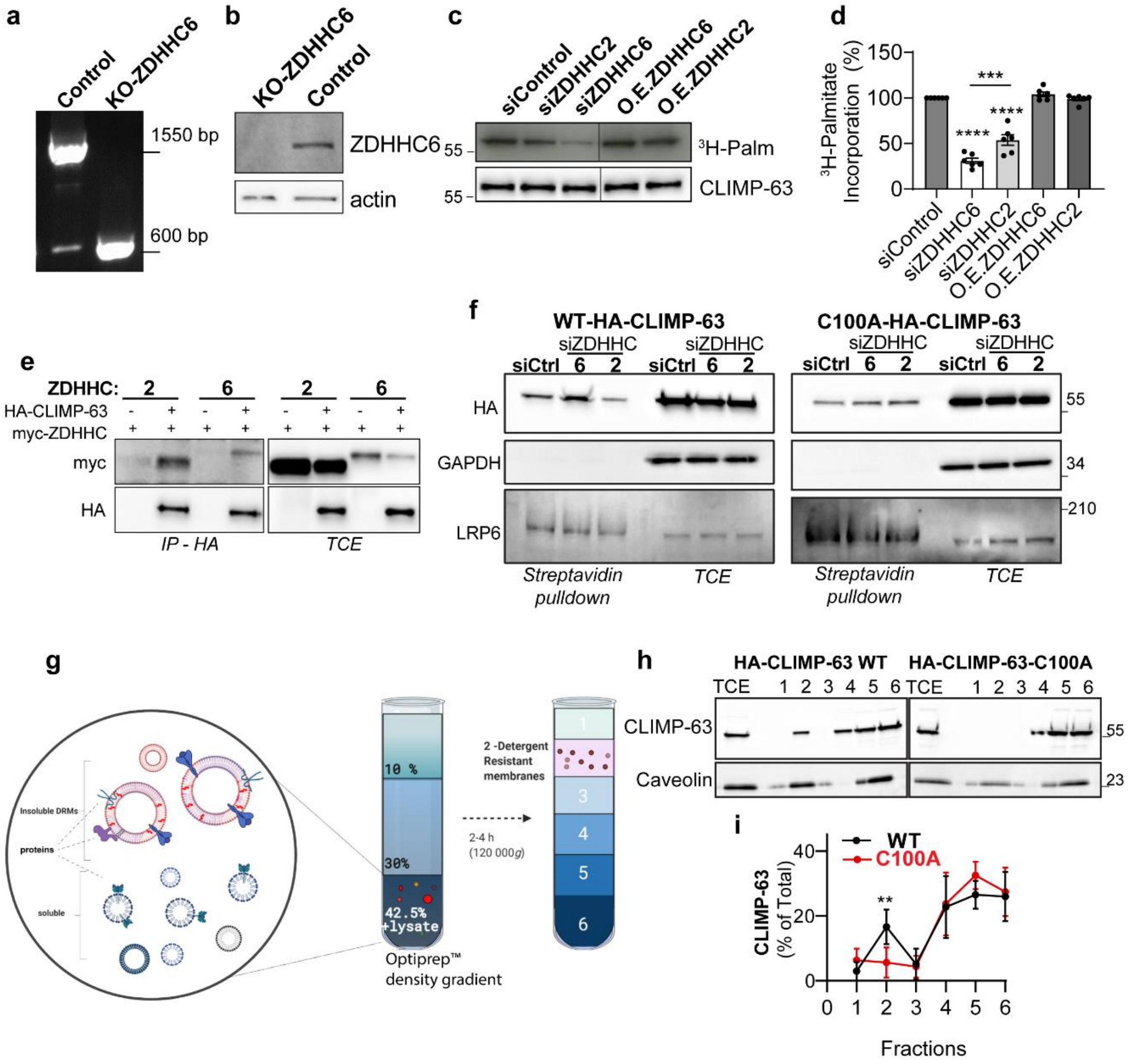

Supplementary Figure 2. Validation of the CRISPR-Cas9 DHHC6 KO HeLa. a. gDNA was extracted, amplified and migrated on agarose gel. The band shift expected for a disruption in exon 2 was confirmed in ZDHHC6 KO cells. **b.** Western blot of ZDHHC6 in KO-DHHC6 versus control HeLa cells. **c.**^{3}H-palmitate metabolic labelling of HeLa cells transfected with control, siZDHHC2, or siZDHHC6 for 72 h, or myc-ZDHHC2 or myc-ZDHHC6 for 24 h. ^{3}H-palmitate present in CLIMP-63 IP fractions were analysed by SDS-PAGE and subsequent autoradiography. Western blot analysis of CLIMP-63 was used to control equivalent protein levels present in IP fractions **d.** Quantification of CLIMP-63 3H-palmitate labelling (results are mean ±SEM, n = 4 biologically independent experiments). ***p=0.0004 and ***p<0.0001. obtained by one-way ANOVA Tukey's multiple comparison **e.** Co-immunoprecipitation of overexpressed HA-CLIMP-63 with ZDHHC2-myc or ZDHHC6-myc in HeLa cells treated with 1 mM of DSP cross-linker for 30 min at 25°C before lysis. **f** Western blot of surface biotinylated proteins and total cell extracts (TCE) from shCLIMP63 cells co-transfected with control, siZDHHC6 or siZDHHC2 and HA-CLIMP-63 (WT or C100A) quantified in figure **2i**. LRP6 and GAPDH are positive and negative controls, respectively. **g.** Schematic representation of DRM isolation – Figure shows homogenised cells extracts in sucrose/optiprep gradients before and after centrifugation. DRMs and associated proteins were harvest from fraction 2. Created with BioRender.com. **h** Western blot of DRM fractionation from shCLIMP-63 cells transfected with HA-CLIMP-63 WT or C100A. Detergent-resistant membranes in fraction 2 are marked by caveolin. Quantification of HA CLIMP-63 in each fraction as a percentage of the sum of all fractions. *p* values compare CLIMP-63 in DRMs (fraction 2), (n=3 biologically independent experiments; ***p*=0.0076, obtained by two-way ANOVA Sydak's multiple comparison.

Supplementary Figure 3

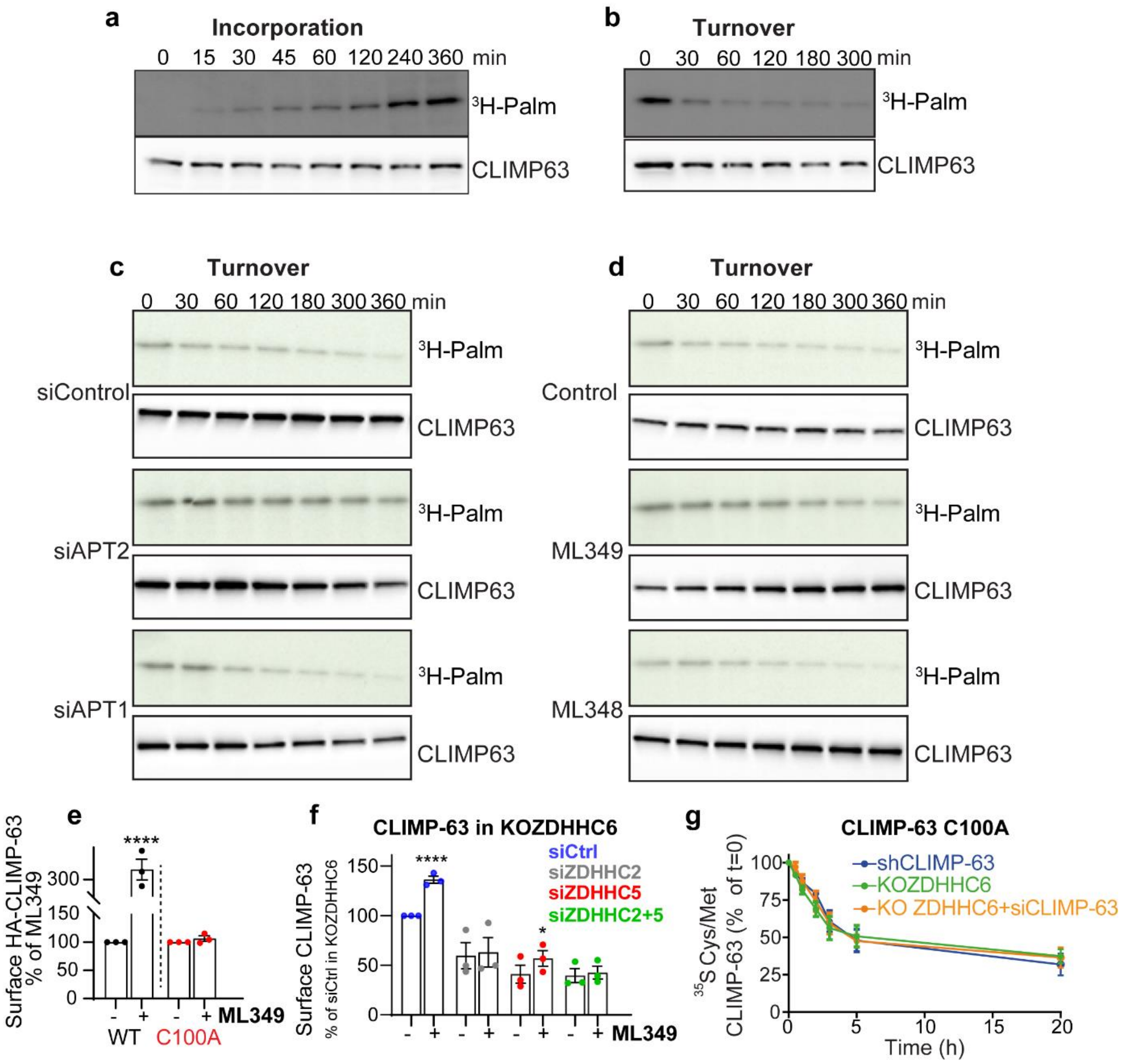

Supplementary Figure 3. ZDHHC6 and APT2 control CLIMP-63 palmitoylation and turnover. a. ^{3}H-palmitate metabolic labelling of HeLa cells for different pulse lengths, quantification shown in Fig. 3a. **b.** ^{3}H-palmitate decay from CLIMP-63 for different chase lengths, quantification shown in Fig. 3b. **c, d.** ^{3}H-palmitate decay from CLIMP-63 in HeLa transfected with indicated siRNA or treated with inhibitors, quantification shown in 3b. **e** Quantification of HA-CLIMP-63 population at the cell surface in shCLIMP-63 cells transfected with siCLIMP-63 and HA-CLIMP-63 (WT or C100A mutant) mock-treated (-) or treated with ML349 for 4 h before surface biotinylation (results are mean±SEM, n=3, *p* values compare surface CLIMP-63±ML349). **f**. same as in **e**, in KO-ZDHHC6 HeLa cells transfected with control, siZDHHC2, siZDHHC5 or siZDHHC2/5. **g. h**. HA-CLIMP-63 (C100A mutant) apparent decay after 20 min pulse of ^{35}S metabolic labelling of the indicated cell samples followed by the indicated periods of chase time. Each sample value was normalized to the initial population (t0) as 100%. Results are mean ± SD. CLIMP-63 C100A mutant was expressed in **g** shCLIMP63, KO-ZDHHC6 or KO-ZDHHC6 cells, all co-transfected with siCLIMP-63. All data is represented as mea ±SEM (e, f) or SD (g) of n=3 independent biological experiments, and all *p* values were obtained by two-way ANOVA, Sydak's multiple comparison (**p*=0.0106, *****p*<0.0001).

Supplementary Figure 4

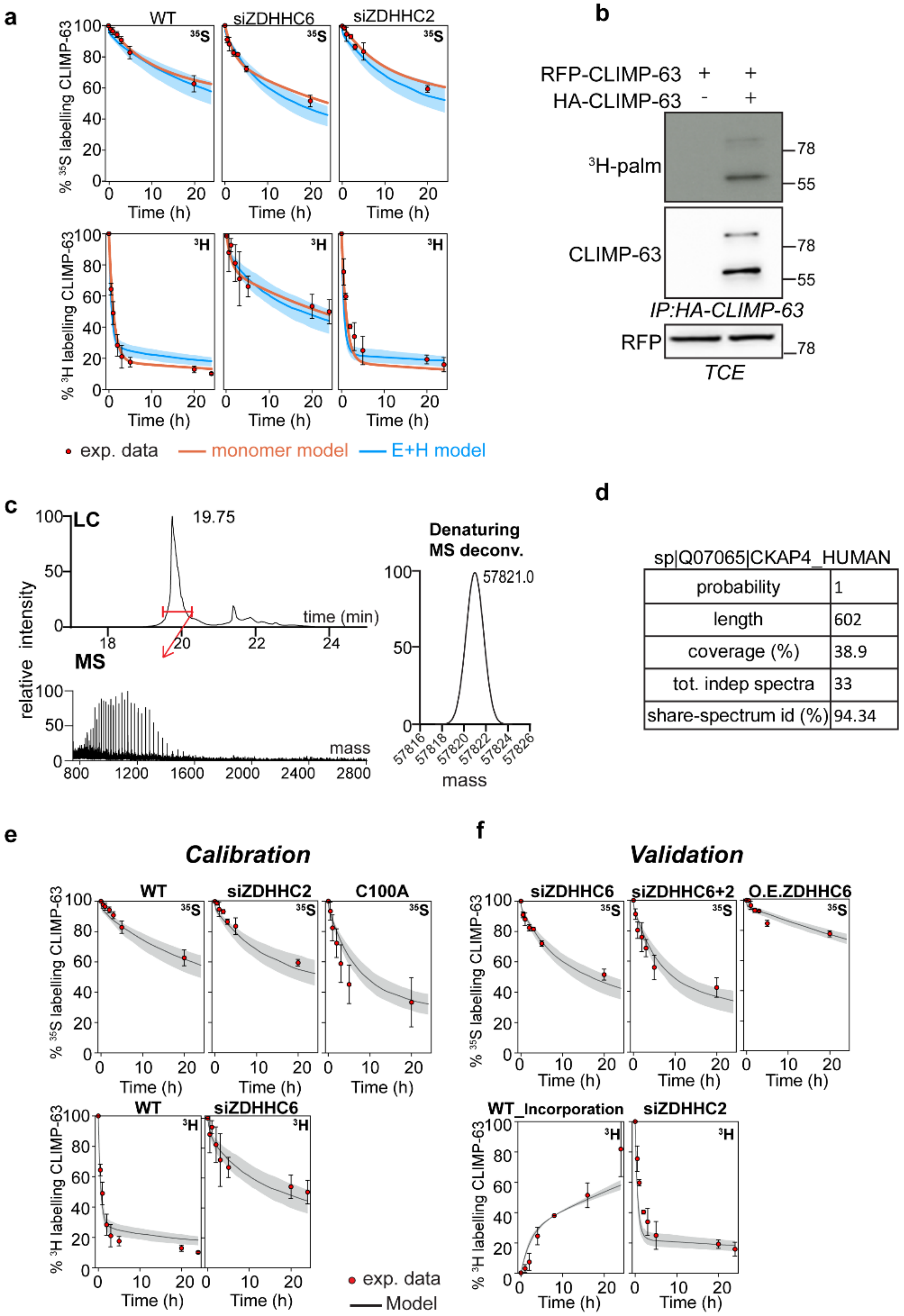

sp|Q07065|CKAP4_HUMAN

probability	1
length	602
coverage (%)	38.9
tot. indep spectra	33
share-spectrum id (%)	94.34

Supplementary Figure 4. Model calibration/validation and CLIMP-63 structural analysis. a. Fit of monomeric (orange) and oligomer (H+E) (blue) model in WT control, ZDHHC2 or ZDHHC6 depletion conditions. **b.** Co-immunoprecipitation and ^{3}H palmitate metabolic labelling of shCLIMP-63 HeLa cells expressing RFP-CLIMP-63 plus or minus HA-CLIMP-63. HA-IP fractions were analysed by Western blot: palmitoylated HA-CLIMP-63 specifically Co-IP with RFP-CLIMP-63 **c.** Intact mass LC-MS analysis under denaturing conditions (Raw and deconvolved spectra) of purified LD-CLIMP-63-FLAG. The analysis of the indicated LC peak (red arrow) revealed a molecular mass correspondent to CLIMP-63 luminal monomers (57821.0 Da). **d.** shotgun proteomic analysis of samples analysed in c and Fig. 4 f-h. Identified sequences, searched within whole human protein database (Swiss-Prot from Uniprot) corresponded to CLIMP-63 (CKAP4) with the indicated output **e. f.** Data used for calibration **(e)** and validation **(f)** of the oligomeric (E+U) model. For all simulation data, Solid lines represent median, shaded intervals the 1st and 3rd quartiles, and red dots experimentally retrieved data points derived from the data represented and detailed in figure 3. Data derived from the simulation of n=100 models and further details of the *in-silico* labelling experiments can be found in the supplementary information – supplementary methods section.

Supplementary Figure 5

a

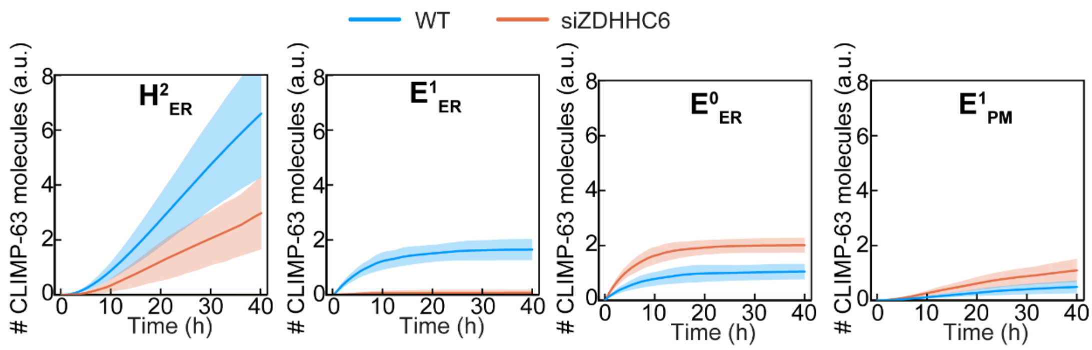

b

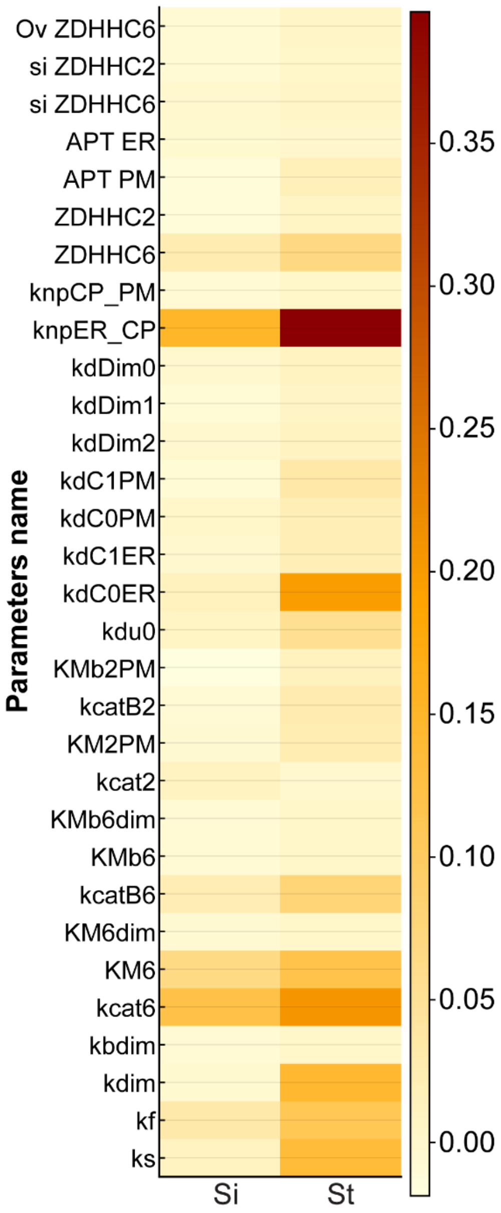

c

ks	Synthesis rate
kf	Folding rate of newly synthesized CLIMP63
kdim	Elementary units (Es) dimerisation rate
kbdim	Higher assemblies (Hs) dissociation rate
kcat6	ZDHHC6 catalytic constant
KM6	Michealis-Menten constant for Es on ZDHHC6
KM6dim	Michealis-Menten constant for Hs on ZDHHC6
kcatB6	APT ER (de-palmitoylation) catalytic constant
KMb6	Michealis-Menten constant for Es on APT ER
KMb6dim	Michealis-Menten constant for Hs on APT ER
kcat2	ZDHHC2 catalytic constant
KM2PM	Michealis-Menten constant for Es on ZDHHC2
kcatB2	APT PM (de-palmitoylation) catalytic constant
KMb2PM	Michealis-Menten constant for Es on APT PM
kdu0	Degradation rate of unfolded CLIMP63
kdC0ER	Degradation rate of unpalmitoylated Es in ER
kdC1ER	Degradation rate of palmitoylated Es in ER
kdC0PM	Degradation rate of unpalmitoylated Es in PM
kdC1PM	Degradation rate of palmitoylated Es in PM
kdDim2	Degradation rate of double palmitoylated Hs (H2)
kdDim1	Degradation rate of single palmitoylated Hs (H1)
kdDim0	Degradation rate of unpalmitoylated Hs (H0)
knpER_CP	Transport constant from ER to CP
knpCP_PM	Transport constant from CP to PM
ZDHHC6	Amount of ZDHHC6
ZDHHC2	Amount of ZDHHC2
APT PM	Amount of APT PM
APT ER	Amount of APT ER
Si ZDHHC6	Silencing factor of ZDHHC6
Si ZDHHC2	Silencing factor of ZDHHC2
Ov ZDHHC6	Overexpression factor of ZDHHC6

Supplementary Figure 5. Validation of CLIMP-63 model a. Evolution in time of the individual CLIMP-63 species simulated as incorporation of ^{35}S label in WT cells (blue) or cells depleted of ZDHHC6 (orange) **b.** Heat map of sobol sensitivity indices for the fitness function used to calibrate the model. Si correspond to 1st order sensitivity indices, and St to the total effect indices. 2^{15} samples of the parameters were taken using a combined Latin Hypercube/Fractional Factorial design with 16 levels. **c** Description of the parameters used in b. Simulation data sets represent the median, and error bars the first and third quartile through the simulation of n=100 models.

Supplementary Figure 6

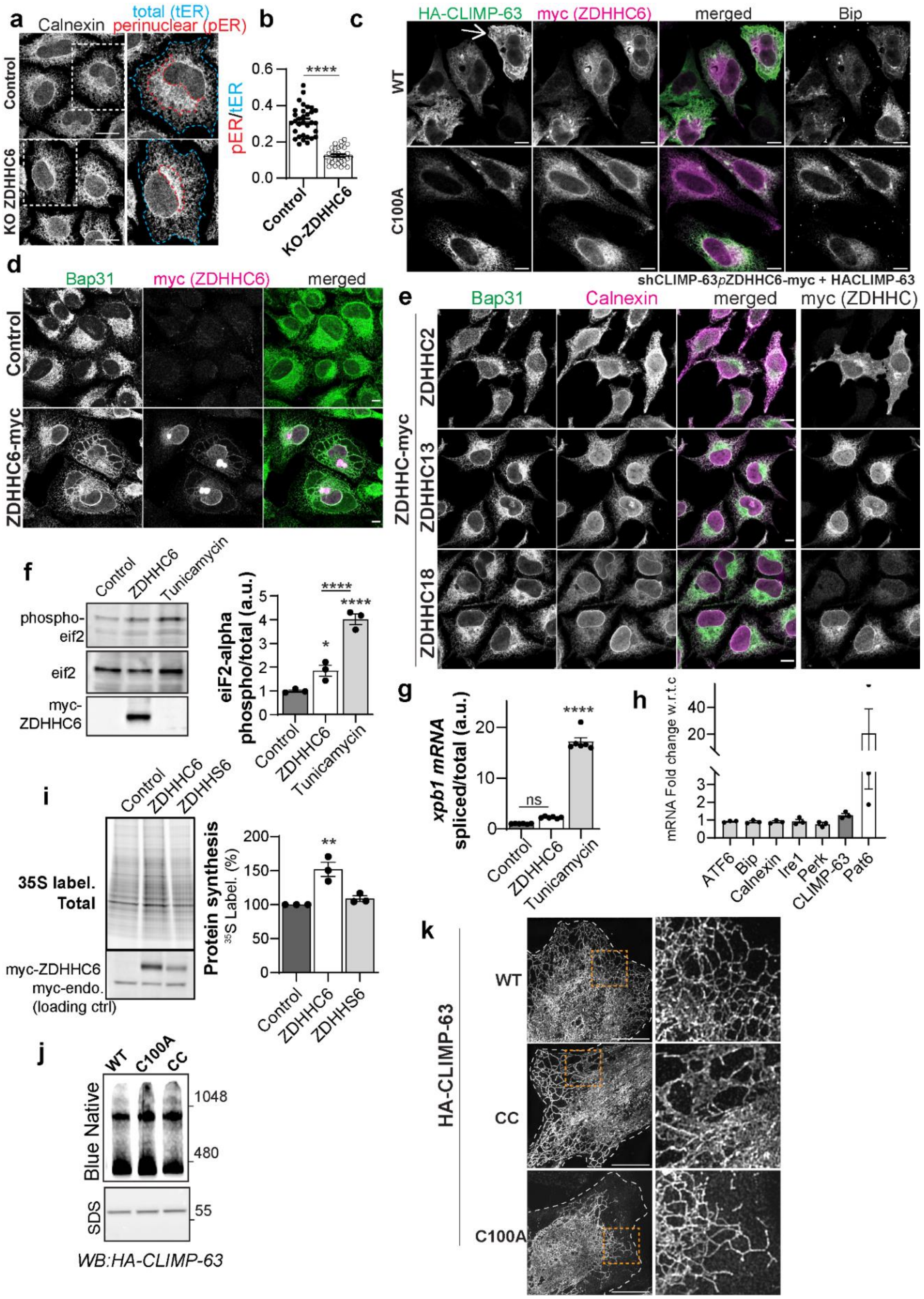

Supplementary Figure 6. CLIMP-63 palmitoylation by ZDHHC6 alters ER morphology. **a.** Confocal immunofluorescence images of HeLa control or ZDHHC6 KO cells immunolabelled for calnexin. **b**. Quantification of the ratio between the area of dense perinuclear ER (pER; red) and total ER (tER; blue). Results are mean±SD of a representative experiment where 32 cells were measured for each condition (****p<0.001 obtained by unpaired, two tailed student's t test). **c**. Airyscan confocal images of shCLIMP-63 HeLa cells expressing ZDHHC6-myc together with HA-CLIMP-63 WT or C100A and immunolabelled for HA (green), myc (magenta) and ER marker Bip (grey). Arrows indicate ZDHHC6-CLIMP-63 WT expressing cells with ER dilation **d.** Confocal immunofluorescence analysis of U2OS cells expressing myc-ZDHHC6 and immunolabelled for myc (magenta) and ER-marker BAP31 (green). **e.** Confocal immunofluorescence images of HeLa cells expressing the indicated ZDHHC enzymes and labelled for myc (grey), Bap31 (green), and calnexin (magenta). **f.** Western blots of total cell extracts (TCE) from HeLa cells transfected with empty plasmids (control), plasmids expressing myc-ZDHHC6 or treated with tunicamicyn (10 µg/ml 4 h). Blots were probed for phospho-eiF2alpha quantified as ratio of total eiF2-alpha. Results are mean ± SEM (n=3), **p*=0.0398, ****p*=0.0005, obtained by one-way ANOVA, Tukey's multiple comparison. **g.** Quantification of the ratio of spliced/total xbp1 mRNA from cells treated as in **f**. Results are mean±SEM (n=6), ****p*<0.0001, obtained by one-way ANOVA, Tukey's multiple comparison. **h.** mRNA fold change of the indicated transcripts in ZDHHC6 transfected cells compared to non-transfected control HeLa cells. results are mean ± SEM (n=3) **i.** ^{35}S metabolic (20 min) of HeLa cells transfected with empty plasmids (control) or plasmids expressing myc- ZDHHC6 or inactive mutant ZDHHS6. Total levels of incorporated ^{35}S were quantified for equivalent protein extracts (loading control-endogenous myc; n=3, ***p*=0.0029 obtained by one-way ANOVA, Tukey's multiple comparison. **j**. Blue Native and SDS-PAGE western blot analysis of shCLIMP-63 HeLa cells expressing HA-CLIMP-63 WT, C100A or CC. **k.** SIM microscopy analysis of shCLIMP-63 cells expressing HA-CLIMP-63 WT, CC or C100A and immunolabelled for HA. Scale bars: 10 µm. Unless otherwise indicated all means were derived from biologically independent experiments.

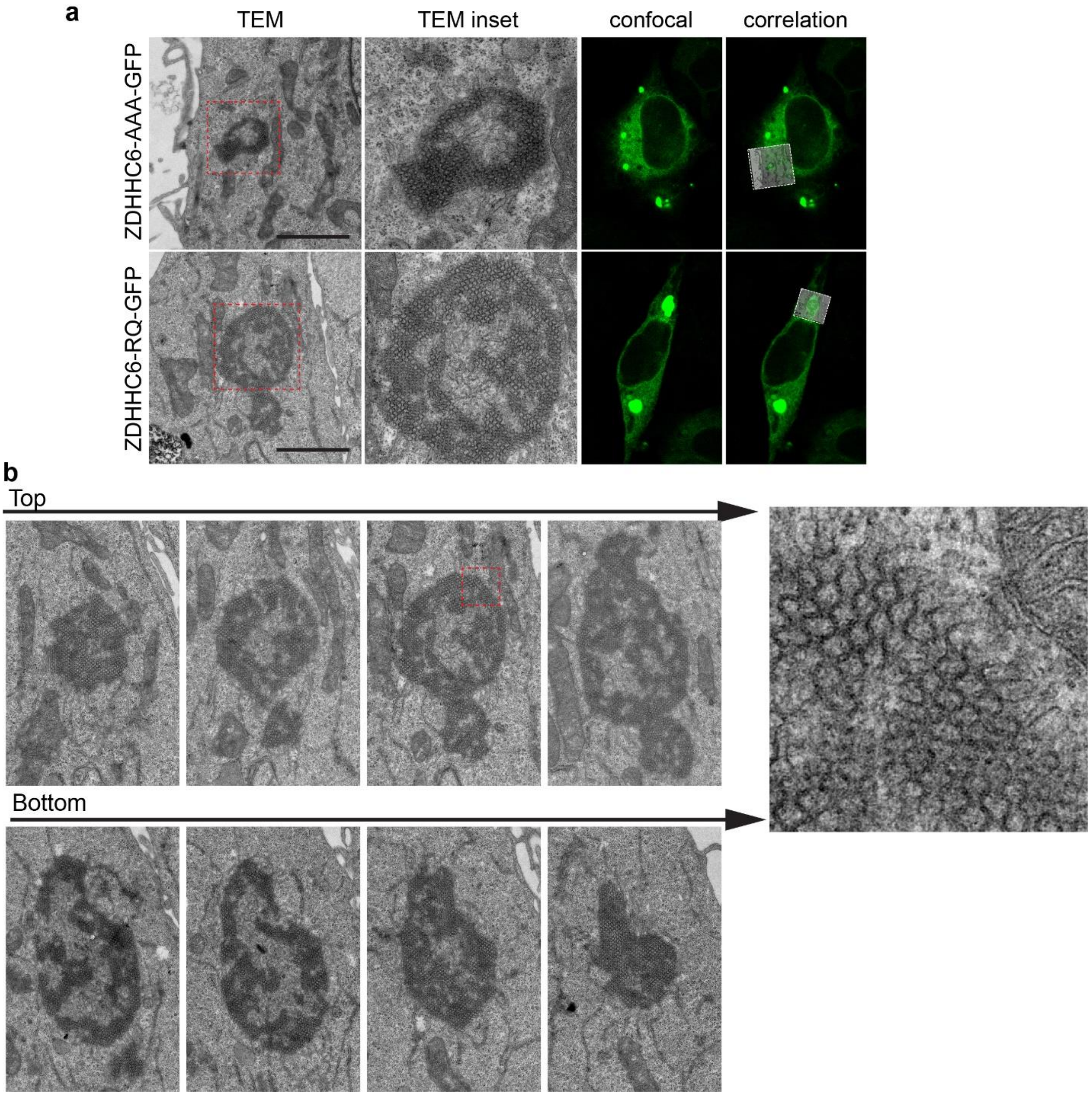

Supplementary Figure 7. CLIMP-63 palmitoylation by ZDHHC6 alter ER morphology. a. Correlative electron microscopy of HeLa cells transfected with ZDHHC6-GFP lacking all three palmitoylation sites (AAA) or ZDHHC6-GFP with a RQ point mutation reducing its catalytic activity. Cells were imaged using an inverted confocal microscope before staining and embedding in resin for transmission electron microscopy processing and analysis. Scale bars: 2 µm. **b.** Serial sections at higher magnification from the top to the bottom of the OSER formed by the ZDHHC6-RQ mutants presented in (a). Higher magnification of the 3rd section from the top of ZDHHC6-RQ-GFP OSER presented in (b). Scale bar: 150 nm.

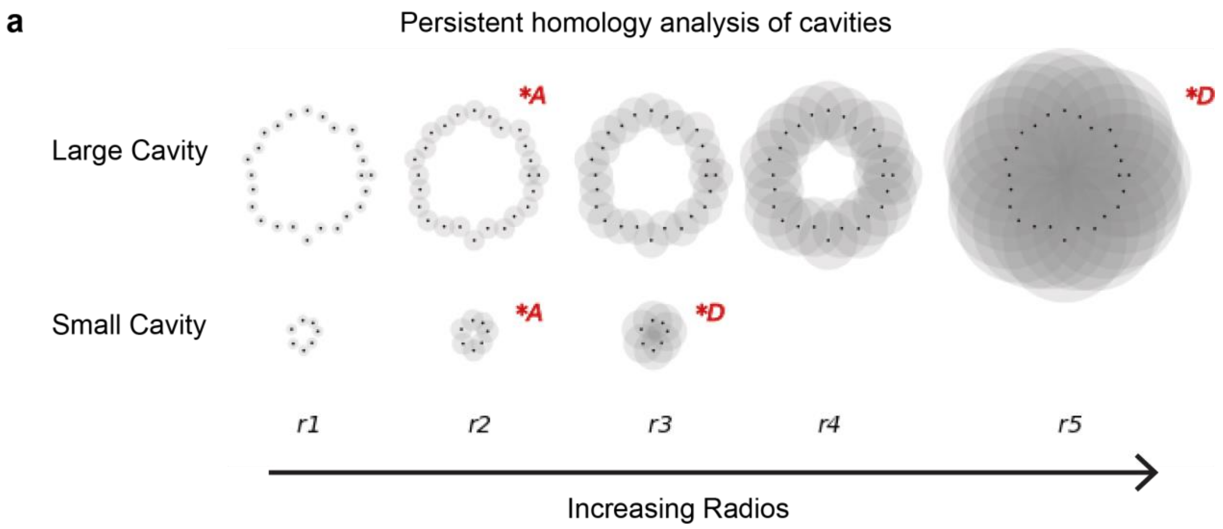

Supplementary Figure 8. Persistent homology analysis of the ER structures shown in Figure 7 A multi-scale view of points sampled from a circle obtained by blowing up disks/balls of growing radii around the points. Degree-1 persistent homology tracks the appearance (*A) of two un-filled loops (e.g. one large and one small) at a scale/radius of around r2, and their disappearance (*D) at a scale/radius of about r5 and r3, respectively.

Extended References

1. Dallavilla, T. *et al.* Model-Driven Understanding of Palmitoylation Dynamics: Regulated Acylation of the Endoplasmic Reticulum Chaperone Calnexin. *PLOS Computational Biology* **12**, e1004774 (2016).
2. Abrami, L. *et al.* Identification and dynamics of the human ZDHHC16-ZDHHC6 palmitoylation cascade. *Elife* **6**, (2017).
3. Segel, L. A. & Slemrod, M. The Quasi-Steady-State Assumption: A Case Study in Perturbation. *SIAM Review* **31**, 446–477 (1989).
4. Segel, L. A. On the validity of the steady state assumption of enzyme kinetics. *Bulletin of Mathematical Biology* **50**, 579–593 (1988).
5. Smith, A. M., Xu, W., Sun, Y., Faeder, J. R. & Marai, G. E. RuleBender: integrated modeling, simulation and visualization for rule-based intracellular biochemistry. *BMC Bioinformatics* **13**, S3 (2012).
6. Hindmarsh, A. C. *et al.* SUNDIALS: Suite of nonlinear and differential/algebraic equation solvers. *ACM Trans. Math. Softw.* **31**, 363–396 (2005).
7. Rackauckas, C. & Nie, Q. DifferentialEquations.jl – A Performant and Feature-Rich Ecosystem for Solving Differential Equations in Julia. *Journal of Open Research Software* **5**, 15 (2017).
8. Hansen, N., Müller, S. D. & Koumoutsakos, P. Reducing the Time Complexity of the Derandomized Evolution Strategy with Covariance Matrix Adaptation (CMA-ES). *Evolutionary Computation* **11**, 1–18 (2003).
9. Loshchilov, I., Schoenauer, M. & Sebag, M. Self-adaptive surrogate-assisted covariance matrix adaptation evolution strategy. in *Proceedings of the 14th annual conference on Genetic and evolutionary computation* 321–328 (Association for Computing Machinery, 2012). doi:10.1145/2330163.2330210.
10. Global Sensitivity Analysis: The Primer | Wiley. *Wiley.com* https://www.wiley.com/en-gb/Global+Sensitivity+Analysis%3A+The+Primer-p-9780470059975.

11. Voronoi, G. Nouvelles applications des paramètres continus à la théorie des formes quadratiques. Premier mémoire. Sur quelques propriétés des formes quadratiques positives parfaites. *Journal für die reine und angewandte Mathematik (Crelles Journal)* **1908**, 97–102 (1908).

12. Edelsbrunner, H. The union of balls and its dual shape. *Discrete Comput Geom* **13**, 415–440 (1995).
