# Supplementary material for: Dynamics of CLIMP-63 S-acylation control ER morphology
Source: Nat Commun. 2023 Jan 17;14:264. doi: 10.1038/s41467-023-35921-6 (PMC9844198; doi:10.1038/s41467-023-35921-6)
Supplement: Supplementary file 3 — Reporting Summary [file 41467_2023_35921_MOESM3_ESM.pdf]

nature portfolio

Corresponding author(s): Francisco Mesquita, Vassily Hatzimanikatis, and F. Gisou van der Goot

Last updated by author(s): Dec 21, 2022

Reporting Summary

Nature Portfolio wishes to improve the reproducibility of the work that we publish. This form provides structure for consistency and transparency in reporting. For further information on Nature Portfolio policies, see our Editorial Policies and the Editorial Policy Checklist.

Statistics

For all statistical analyses, confirm that the following items are present in the figure legend, table legend, main text, or Methods section.

n/a	Confirmed	
☐	☒	The exact sample size (*n*) for each experimental group/condition, given as a discrete number and unit of measurement
☐	☒	A statement on whether measurements were taken from distinct samples or whether the same sample was measured repeatedly
☐	☒	The statistical test(s) used AND whether they are one- or two-sided<br>*Only common tests should be described solely by name; describe more complex techniques in the Methods section.*
☐	☒	A description of all covariates tested
☐	☒	A description of any assumptions or corrections, such as tests of normality and adjustment for multiple comparisons
☐	☒	A full description of the statistical parameters including central tendency (e.g. means) or other basic estimates (e.g. regression coefficient) AND variation (e.g. standard deviation) or associated estimates of uncertainty (e.g. confidence intervals)
☐	☒	For null hypothesis testing, the test statistic (e.g. *F*, *t*, *r*) with confidence intervals, effect sizes, degrees of freedom and *P* value noted<br>*Give P values as exact values whenever suitable.*
☒	☐	For Bayesian analysis, information on the choice of priors and Markov chain Monte Carlo settings
☒	☐	For hierarchical and complex designs, identification of the appropriate level for tests and full reporting of outcomes
☒	☐	Estimates of effect sizes (e.g. Cohen's *d*, Pearson's *r*), indicating how they were calculated

Our web collection on statistics for biologists contains articles on many of the points above.

Software and code

Policy information about availability of computer code

Data collection

Microscopy Microscopy images were acquired using ZEN 2009, ZEN Blue ver. 3.4.91, NIS Elements with JOBS. Western blot images collected with fusion solo 6S edge. EM final images were precisely aligned using the StackReg algorithm (56) in ImageJ, and the ER, mitochondria, nuclear membrane, and cell membrane were segmented using the Microscopy Image Browser software. Computations for persistent homology were done GUDHI software2.0.0: @book{gudhi
, title = "{GUDHI} User and Reference Manual"
, author = "{The GUDHI Project}"
, publisher = "{GUDHI Editorial Board}"
, year = 2015
}
ad-hoc scripts and code were used for analysis rendering and visualisation of persistence diagrams.
Computational Modeling:
In order to automatically generate an ODE (Ordinary Differential Equation) system, rule-based modeling with RuleBender was used.
In order to solve the ODE system the Sundials interface of Differential Equations.jl was used within Julia 0.6. All further details concerning the computational modelling are detailied in Supplementary information.

Data analysis	Data visualization and statistical analysis were performed using GraphPad-Prism_9. Fluorescent image processing and western blot analysis - ImageJ-win6. Intact mass LC-MS data analysis was performed with Protein Deconvolution 4.0 (Thermo Fischer Scientific, Sunnyvale, CA, USA) using Respect algorithm. Analysis was performed using Trans-Proteomic Pipeline software (TPP, Institute for Systems Biology, Seattle Proteome Center) using Tandem pipeline with X-Tandem search engine; Rendering and visualization of focus ion bean final mesh models were performed with Blender software (www.blender.org).

For manuscripts utilizing custom algorithms or software that are central to the research but not yet described in published literature, software must be made available to editors and reviewers. We strongly encourage code deposition in a community repository (e.g. GitHub). See the Nature Portfolio guidelines for submitting code & software for further information.

Data

Policy information about availability of data

All manuscripts must include a data availability statement. This statement should provide the following information, where applicable:

- Accession codes, unique identifiers, or web links for publicly available datasets
- A description of any restrictions on data availability
- For clinical datasets or third party data, please ensure that the statement adheres to our policy

The authors declare that all data supporting the findings of this study are available within the paper, the Supplementary Information and Supplementary Data Source files. Other specific enquiries and data sets are available upon reasonable request.
Further details on materials, methods and computational analysis used in this study can be found in Supplementary Information.
The Mass Spectrometry data sets (processed and RAW data) generated in this study have been deposited in the Mendeley data database and are available at: Mesquita, Francisco (2022), "Sandoz, P._etal_2022_MassSpecDataSource", Mendeley Data, V1, doi: 10.17632/cx28dr9t22.1

Human research participants

Policy information about studies involving human research participants and Sex and Gender in Research.

Reporting on sex and gender	NA
Population characteristics	NA
Recruitment	NA
Ethics oversight	NA

Note that full information on the approval of the study protocol must also be provided in the manuscript.

Field-specific reporting

Please select the one below that is the best fit for your research. If you are not sure, read the appropriate sections before making your selection.

☒ Life sciences ☐ Behavioural & social sciences ☐ Ecological, evolutionary & environmental sciences

For a reference copy of the document with all sections, see nature.com/documents/nr-reporting-summary-flat.pdf

Life sciences study design

All studies must disclose on these points even when the disclosure is negative.

Sample size	All sample sizes and correspondent statistical comparisons are indicated in figure legends or methods sections. For microscopy qualitative quantifications the number of analyzed cells per set is indicated. For all experiments we chose to analyse three or more biological independent samples/assays.
Data exclusions	no data was excluded
Replication	For all experiments we chose to analyses three or more biological independent assays. All replicates were included in the analysis, or displayed equivalent results (such as blots)
Randomization	non applicable to this study because human bias were not present in the presented measurements (all within subject experiments)
Blinding	Unmarked microscopy slides were used for qualitative quantification whenever suited (Fig 6abc, i and j). The remaining measurements were not subjected to bias.

Reporting for specific materials, systems and methods

We require information from authors about some types of materials, experimental systems and methods used in many studies. Here, indicate whether each material, system or method listed is relevant to your study. If you are not sure if a list item applies to your research, read the appropriate section before selecting a response.

Materials & experimental systems

n/a	Involved in the study
☐	☒ Antibodies
☐	☒ Eukaryotic cell lines
☒	☐ Palaeontology and archaeology
☒	☐ Animals and other organisms
☒	☐ Clinical data
☒	☐ Dual use research of concern

Methods

n/a	Involved in the study
☒	☐ ChIP-seq
☒	☐ Flow cytometry
☒	☐ MRI-based neuroimaging

Antibodies

Antibodies used

myc (RRID:AB_2537024) and Bap31 (RRID:AB_325095) antibodies were from Thermo Fisher (US). Anti-CLIMP-63 were either from Alexis/ENZO (G1/296, CH, RRID:AB_2051140) or Bethyl Laboratories (A302, RRID:AB_1731083). anti-LRP6 were also from Bethyl Laboratories (US), (RRID:AB_21393299). Anti-calreticulin (RRID:AB_1267911), anti-Spastin (RRID:AB_2042945) and anti-BiP (RRID:AB_880312) were from Abcam (UK). Anti-tubulin (RRID:AB_477579), anti-GAPDH (RRID:AB_2533438), anti-ZDHHC6 (RRID:AB_2304658), anti-FLAG (RRID:AB_439685), anti-LPXN (RRID:AB_1853250), anti-Caveolin1(RRID:AB_476842) and anti-transferrin receptor (RRID:AB_86623) were from Sigma (US). Anti-actin was from Millipore (US) (RRID:AB_2223041). Anti-HA was from BioLegend (US) (RRID:AB_2563418). Anti-GFP (RRID:AB_2336883) and anti-RFP (RRID:AB_ 2336063) were from Roche (CH). Anti-calnexin was previously described 1 n provided by Dr. M. Molinari. Anti-TRAPα was provided by Dr. R. Hegde. Anti-HA-HRP conjugated was from Roche (CH) (RRID:AB_39091). Rabbit anti-EIF2alpha (Cell Signalling #9722), rabbit anti-eIF2 alpha (Phospho-Ser51) (Biorbyt #orb5998, RRID:AB_10928244). Secondaries: Anti mouse A-11029, A-10037, A-31571, Anti-rabbit, A-21206, A-10042, A-31573, all Thermofisher Scientific

Validation

All antibodies used in this study have been commercially validated or, when applicable, validated using siRNA to the targeted proteins.

Eukaryotic cell lines

Policy information about cell lines and Sex and Gender in Research

Cell line source(s)

RPE-1 cells, CRL4000 - ATCC; Hela cells CCL-2, ATCC; HEK293T, CRL- 3216. Parental cell lines were used to engineer HeLa shCLIMP-63 and HeLa CRISPR-KO-ZDHHC6 as described in method section Hek293T, CRL-3216, ATCC

Authentication

All parental cell lines were commercially acquired and validated

Mycoplasma contamination

Cells were confirmed mycoplasma negative as tested on a trimestral basis using the MycoProbe Mycoplasma Detection Kit CUL001B

Commonly misidentified lines (See ICLAC register)

NA No misidentified cell lines were used

Palaeontology and Archaeology

Specimen provenance

NA

Specimen deposition

NA

Dating methods

NA

☐ Tick this box to confirm that the raw and calibrated dates are available in the paper or in Supplementary Information.

Ethics oversight

NA

Note that full information on the approval of the study protocol must also be provided in the manuscript.

Animals and other research organisms

Policy information about studies involving animals; ARRIVE guidelines recommended for reporting animal research, and Sex and Gender in Research

Laboratory animals

no animals were used in this study

Wild animals

NA

Reporting on sex

NA

Field-collected samples	NA
Ethics oversight	*Identify the organization(s) that approved or provided guidance on the study protocol, OR state that no ethical approval or guidance was required and explain why not.*

Note that full information on the approval of the study protocol must also be provided in the manuscript.

Clinical data

Policy information about clinical studies

All manuscripts should comply with the ICMJE guidelines for publication of clinical research and a completed CONSORT checklist must be included with all submissions.

Clinical trial registration	NA
Study protocol	NA
Data collection	NA
Outcomes	NA

Dual use research of concern

Policy information about dual use research of concern

Hazards

Could the accidental, deliberate or reckless misuse of agents or technologies generated in the work, or the application of information presented in the manuscript, pose a threat to:

No	Yes	
☒	☐	Public health
☒	☐	National security
☒	☐	Crops and/or livestock
☒	☐	Ecosystems
☒	☐	Any other significant area

Experiments of concern

Does the work involve any of these experiments of concern:

No	Yes	
☒	☐	Demonstrate how to render a vaccine ineffective
☒	☐	Confer resistance to therapeutically useful antibiotics or antiviral agents
☒	☐	Enhance the virulence of a pathogen or render a nonpathogen virulent
☒	☐	Increase transmissibility of a pathogen
☒	☐	Alter the host range of a pathogen
☒	☐	Enable evasion of diagnostic/detection modalities
☒	☐	Enable the weaponization of a biological agent or toxin
☒	☐	Any other potentially harmful combination of experiments and agents

ChIP-seq

Data deposition

☐ Confirm that both raw and final processed data have been deposited in a public database such as GEO.

☐ Confirm that you have deposited or provided access to graph files (e.g. BED files) for the called peaks.

Data access links<br>*May remain private before publication.*	NA
Files in database submission	NA
Genome browser session<br>(e.g. UCSC)	NA

Methodology

Replicates	NA
Sequencing depth	NA
Antibodies	NA
Peak calling parameters	NA
Data quality	NA
Software	NA

Flow Cytometry

Plots

Confirm that:

☐ The axis labels state the marker and fluorochrome used (e.g. CD4-FITC).

☐ The axis scales are clearly visible. Include numbers along axes only for bottom left plot of group (a 'group' is an analysis of identical markers).

☐ All plots are contour plots with outliers or pseudocolor plots.

☐ A numerical value for number of cells or percentage (with statistics) is provided.

Methodology

Sample preparation	NA
Instrument	NA
Software	NA
Cell population abundance	NA
Gating strategy	NA

☐ Tick this box to confirm that a figure exemplifying the gating strategy is provided in the Supplementary Information.

Magnetic resonance imaging

Experimental design

Design type	NA
Design specifications	NA
Behavioral performance measures	NA

Acquisition

Imaging type(s)	NA
Field strength	NA
Sequence & imaging parameters	NA
Area of acquisition	NA

Diffusion MRI ☐ Used ☐ Not used

Preprocessing

Preprocessing software	NA
Normalization	NA

Normalization template	NA
Noise and artifact removal	NA
Volume censoring	NA

Statistical modeling & inference

Model type and settings	NA
Effect(s) tested	NA

Specify type of analysis: ☐ Whole brain ☐ ROI-based ☐ Both

Statistic type for inference (See Eklund et al. 2016)	NA
Correction	NA

Models & analysis

n/a	Involved in the study
☐	☐ Functional and/or effective connectivity
☐	☐ Graph analysis
☐	☐ Multivariate modeling or predictive analysis

Functional and/or effective connectivity	NA
Graph analysis	NA
Multivariate modeling and predictive analysis	NA
