# Supplementary material for: Dynamics of CLIMP-63 S-acylation control ER morphology
Source: Nat Commun. 2023 Jan 17;14:264. doi: 10.1038/s41467-023-35921-6 (PMC9844198; doi:10.1038/s41467-023-35921-6)

Figure blots data source (Sandoz. P, et al 2022)

Dynamics of CLIMP-63 S-acylation control ER morphology

Fig 1a

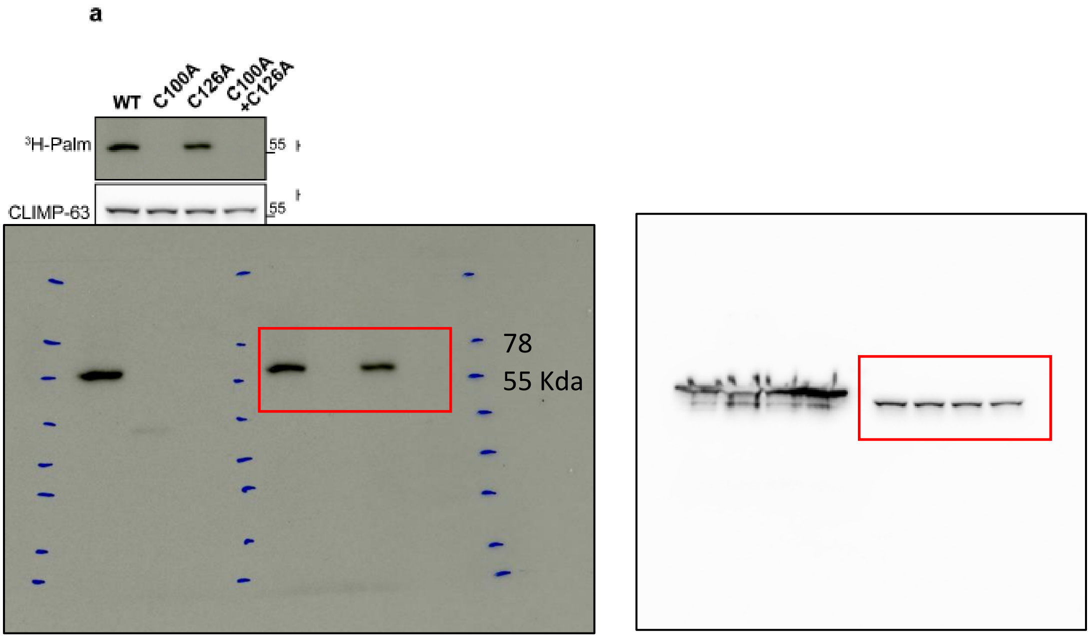

Autoradiography

WB anti- climp63

Fig 1b

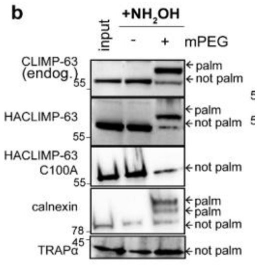

Figure 1b: pegylation

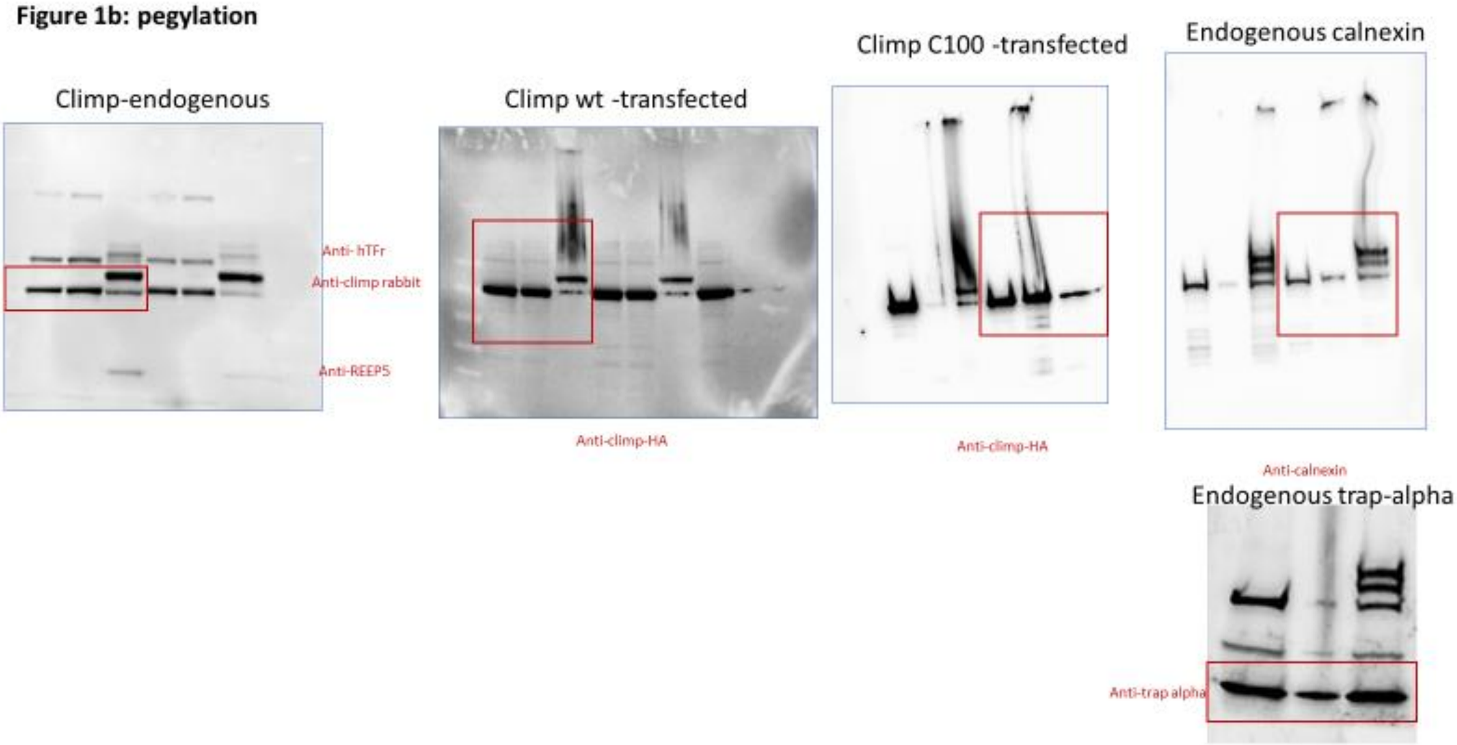

Fig 1c

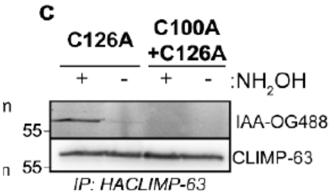

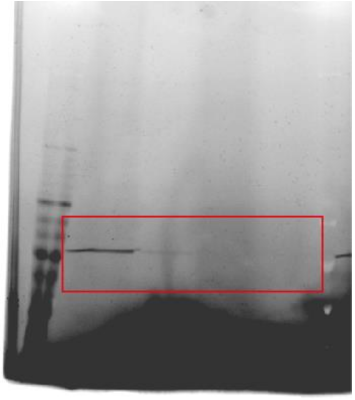

IAA-OG488

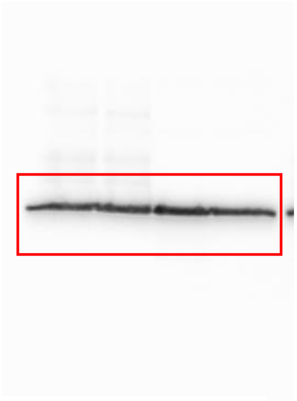

WB:CLIMP-63

Fig 1d

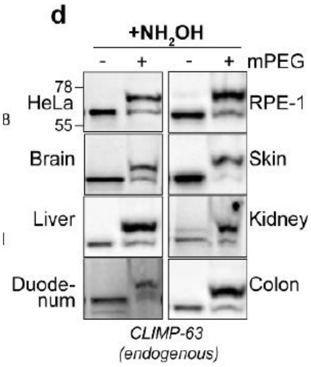

Figure 1d: pegylation

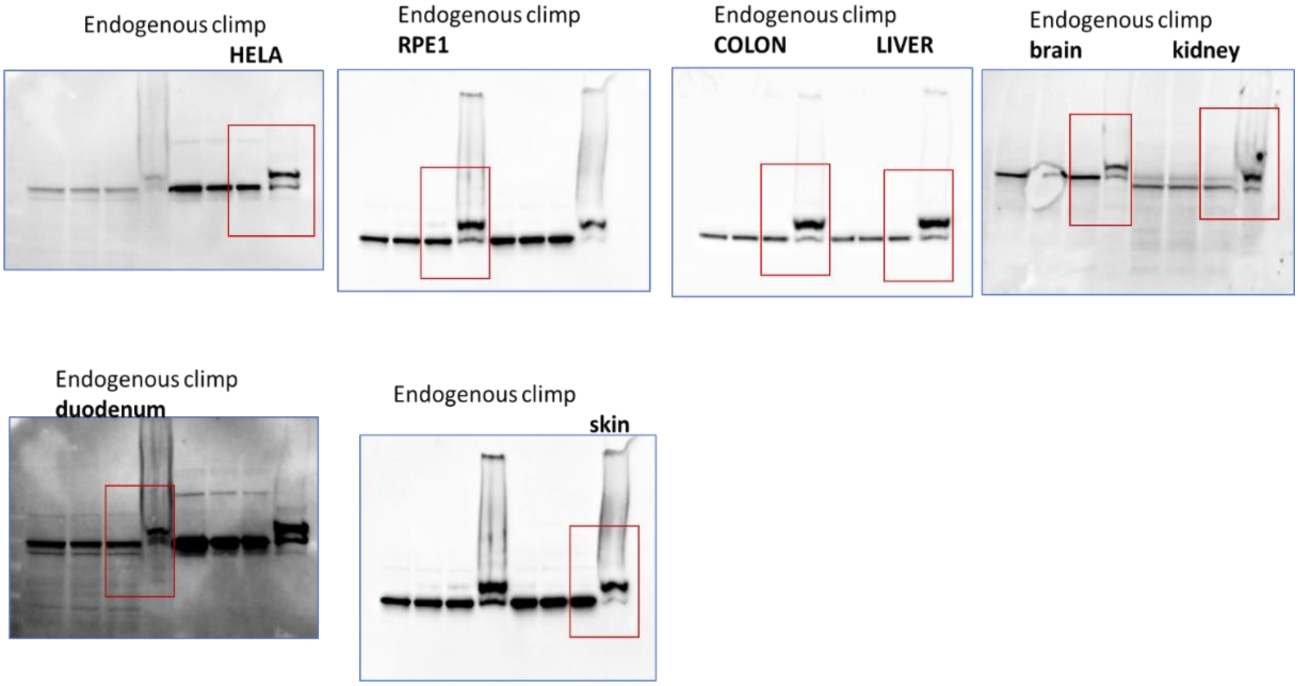

Fig 1e

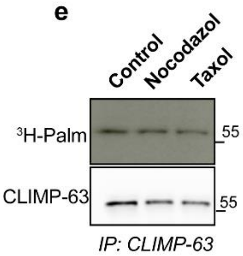

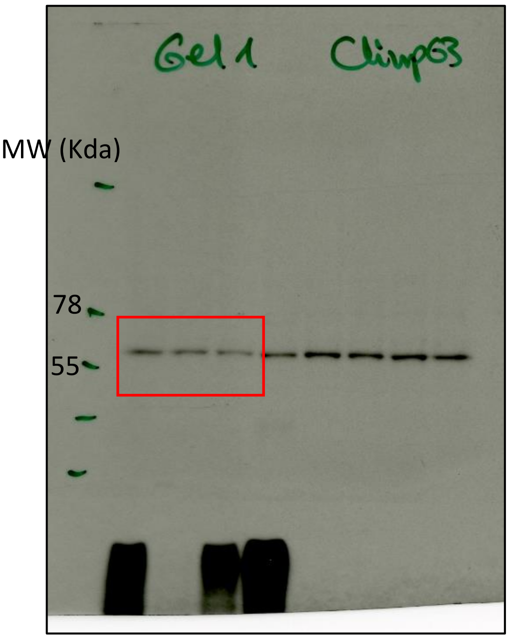

Autoradiography

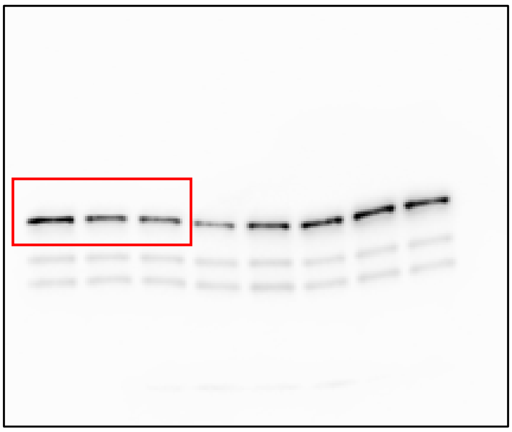

WB anti- climp63

Fig 1f

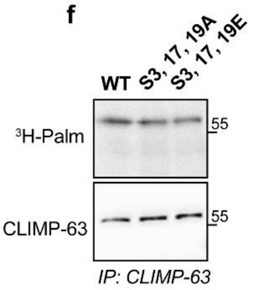

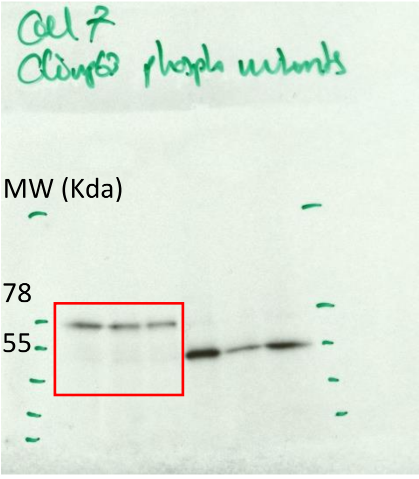

Autoradiography

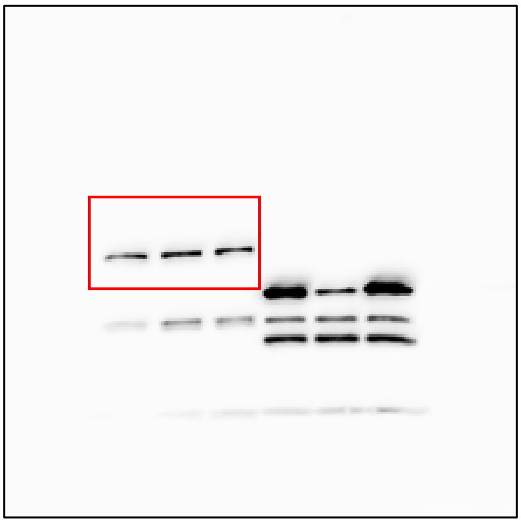

WB anti- climp63

Fig S1a

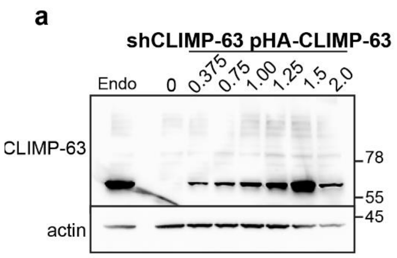

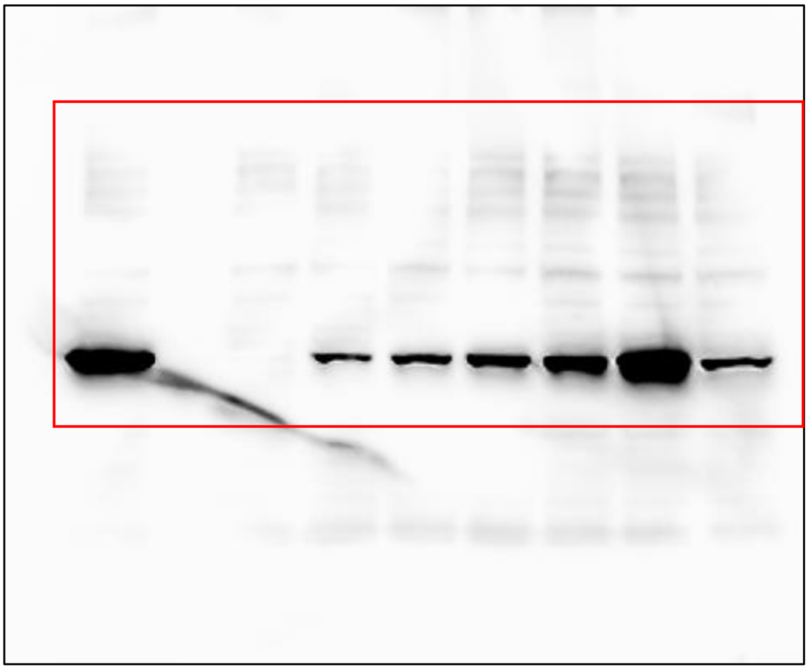

WB anti- climp63

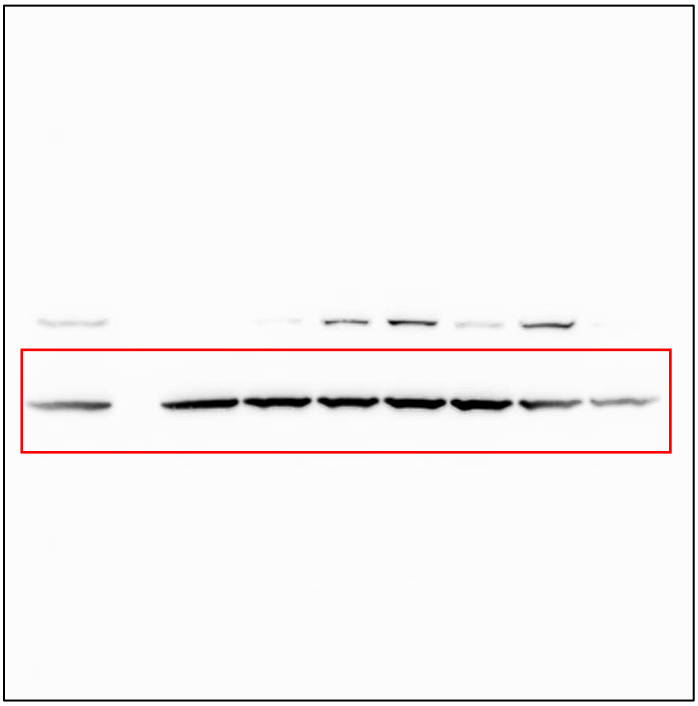

WB anti- actin

Fig S1c

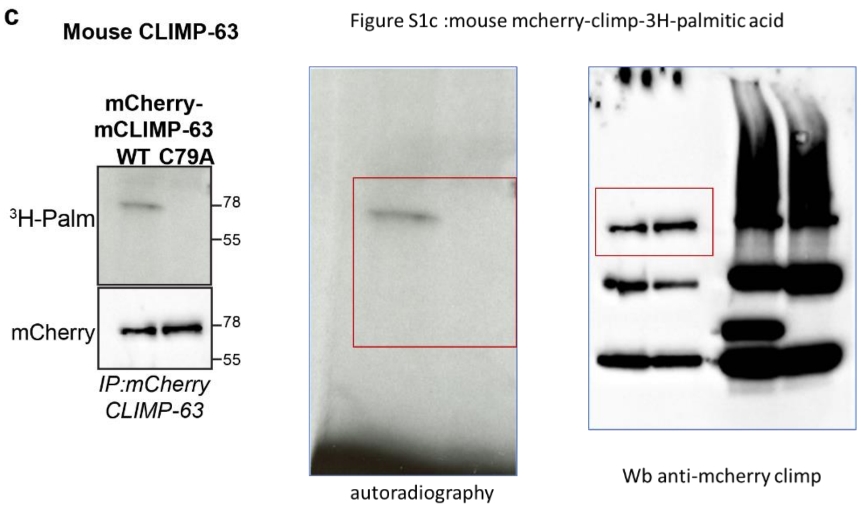

Fig S1d:

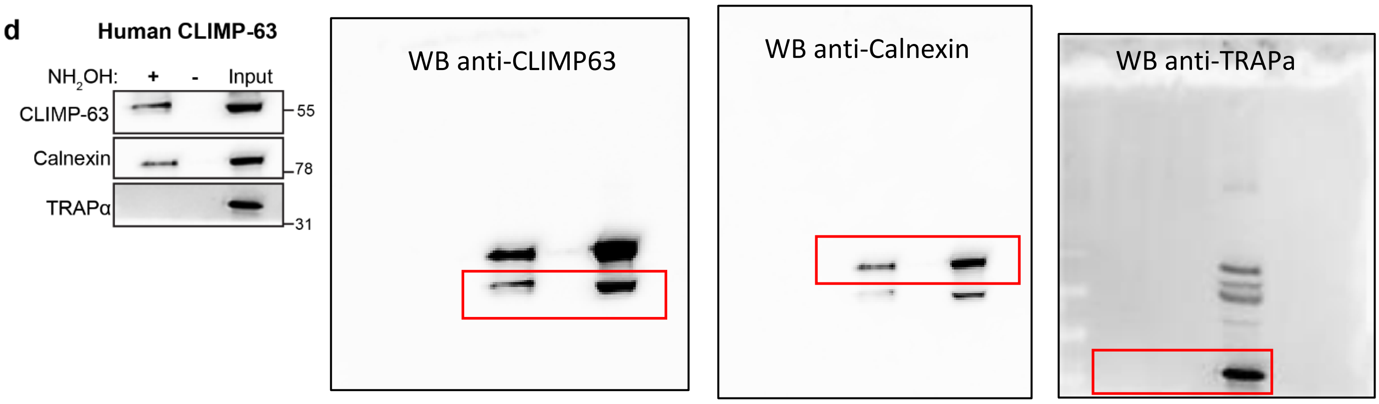

Fig S1e

e

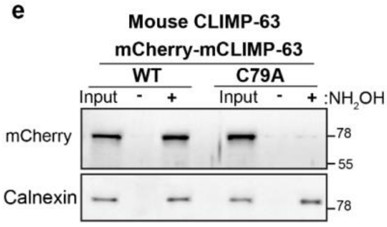

Figure S1e :mouse mcherry-climp-3H-palmitic acid

Wb anti-mcherry climp

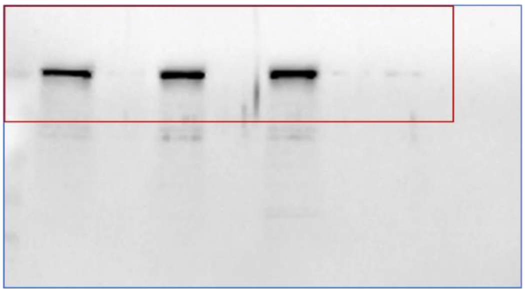

Wb anti-calnexin

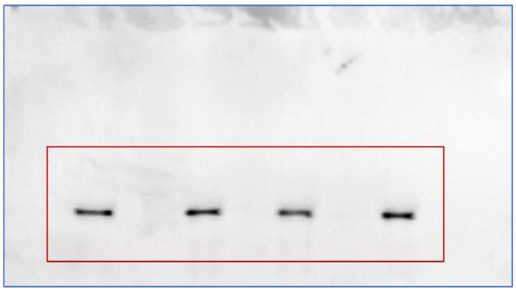

Fig 2a

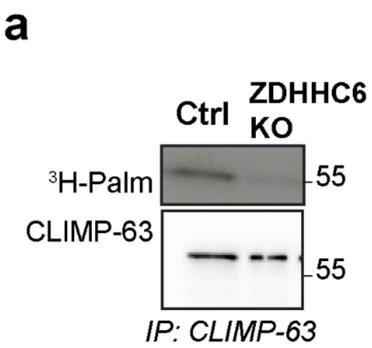

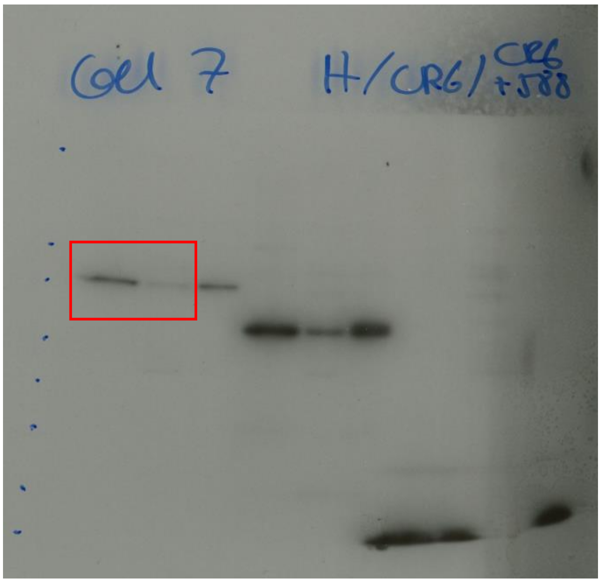

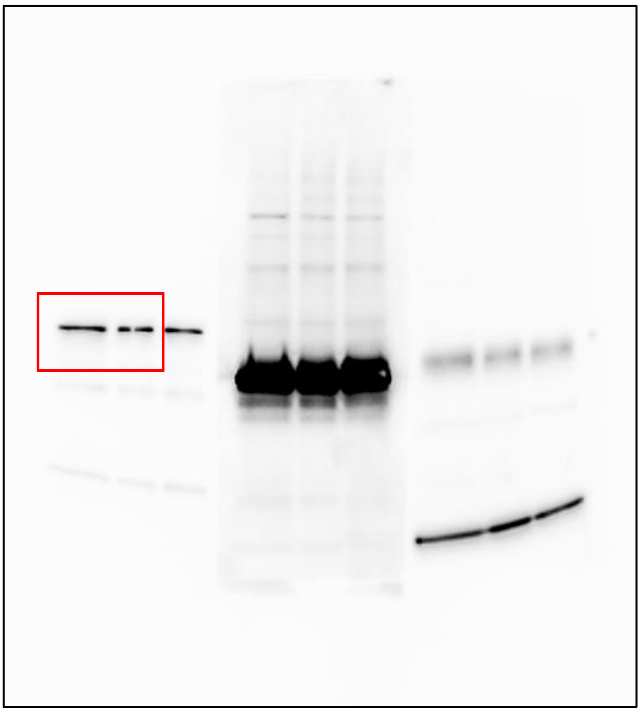

Fig 2b

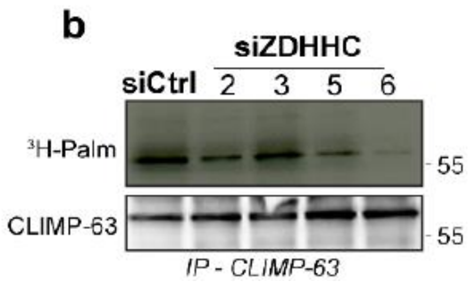

Figure 2b

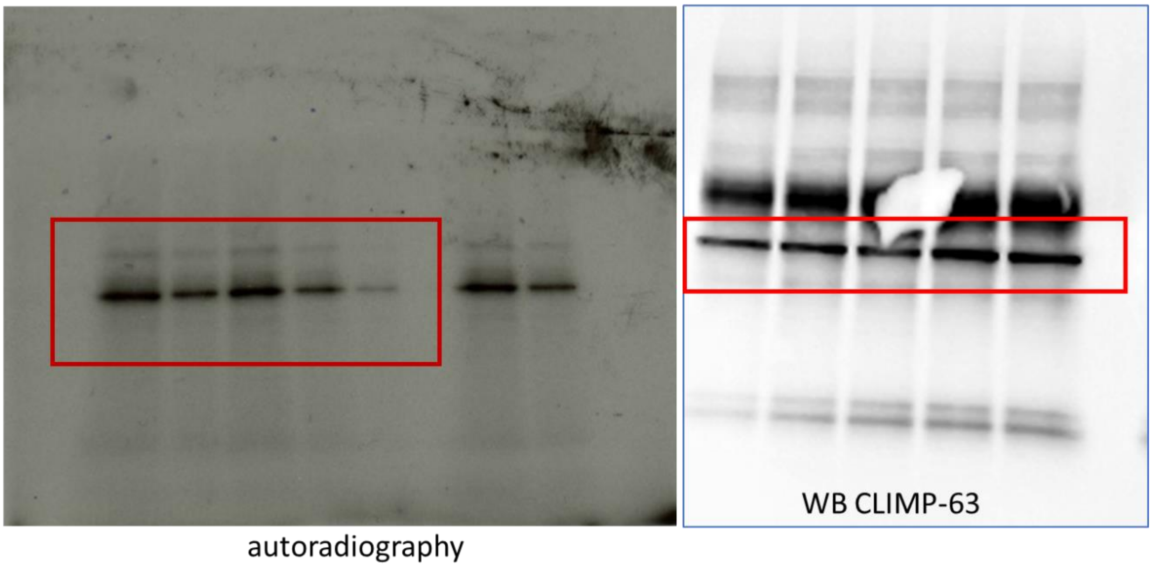

Fig 2f

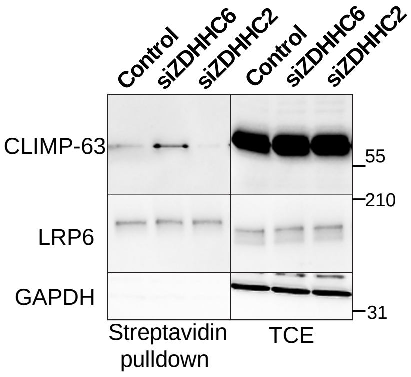
Control
siZDHHC6
siZDHHC2
Control
siZDHHC6
siZDHHC2
CLIMP-63
LRP6
GAPDH
55
210
31
Streptavidin pulldown
TCE

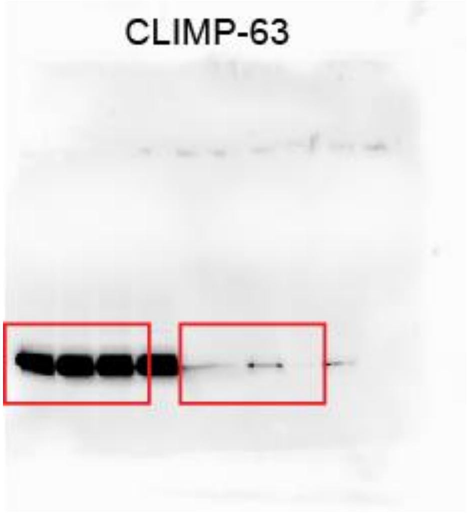
CLIMP-63

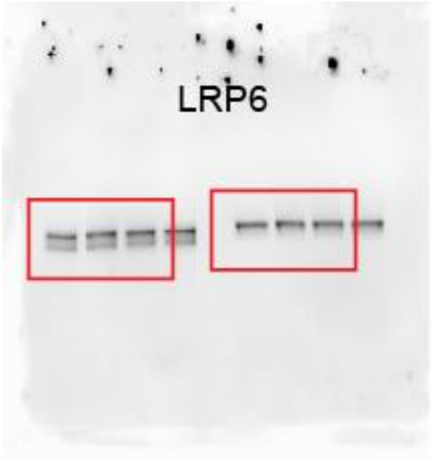
LRP6

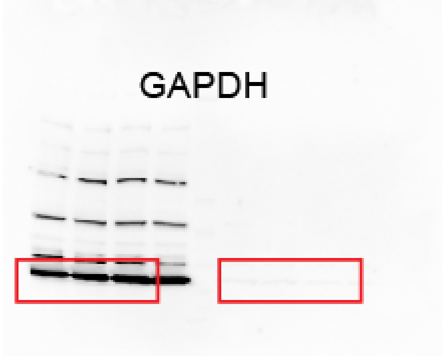
GAPDH

Fig 2h

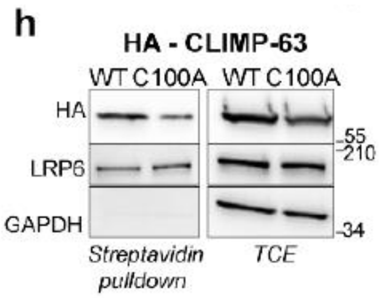
h
HA - CLIMP-63
WT C100A WT C100A
HA
LRP6
GAPDH
55
210
34
Streptavidin pulldown
TCE

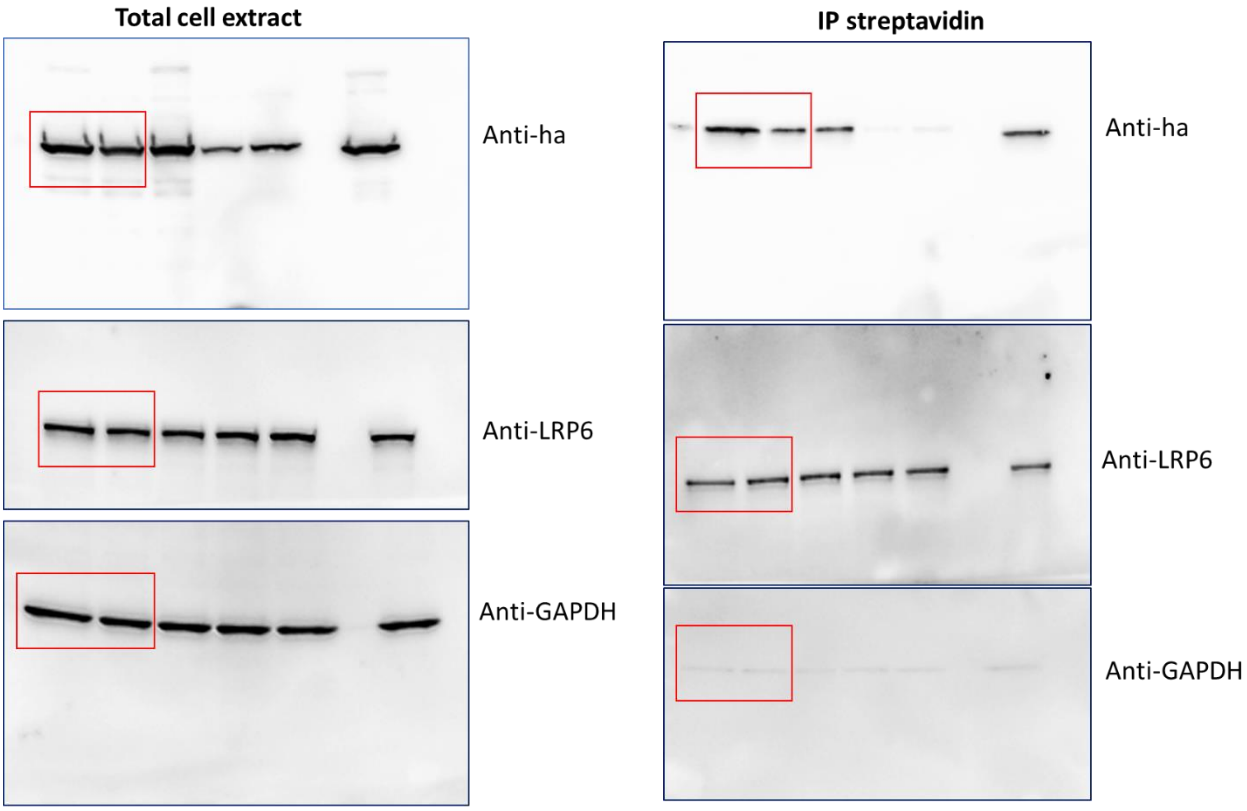
Total cell extract
IP streptavidin
Anti-ha
Anti-LRP6
Anti-GAPDH
Anti-ha
Anti-LRP6
Anti-GAPDH

Fig 2j

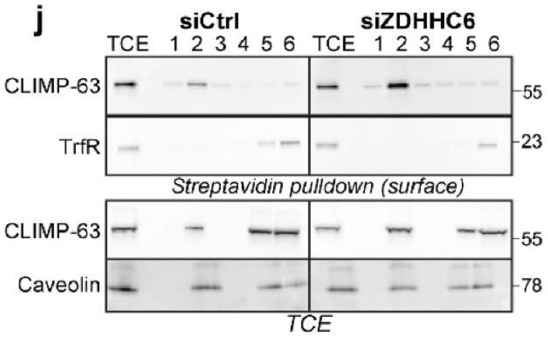

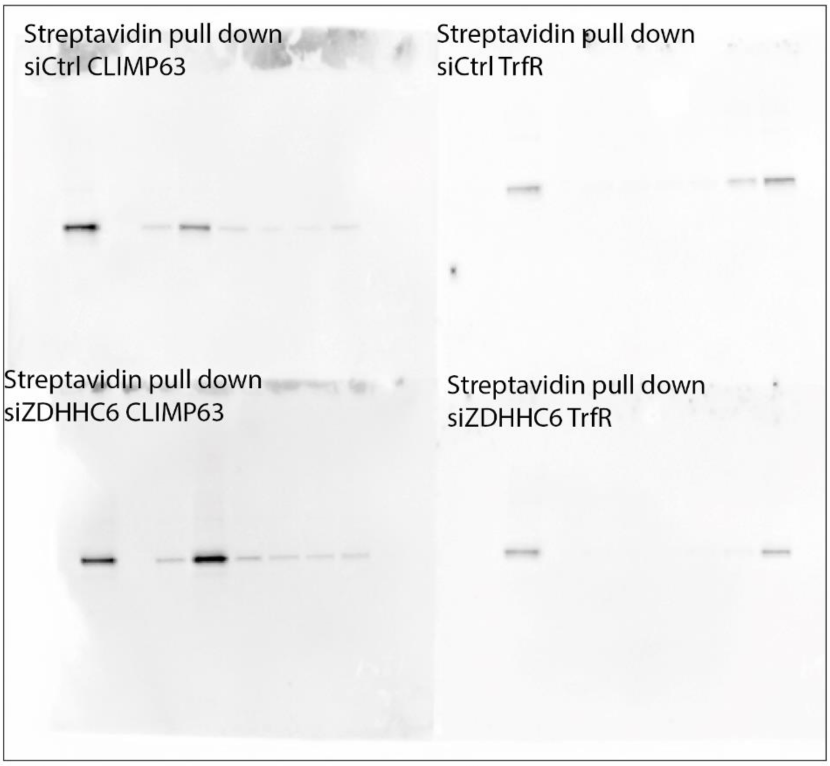

TCE
siCtrl CLIMP63

TCE
siZDHHC6 CLIMP63

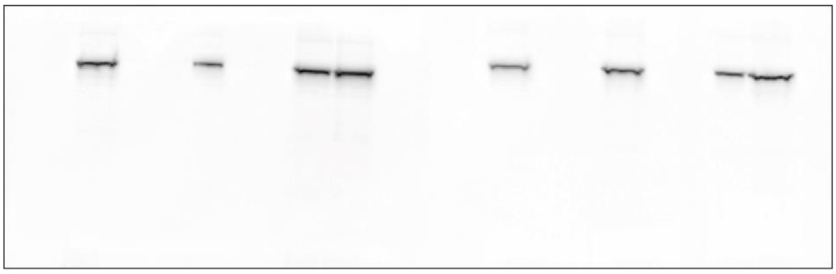

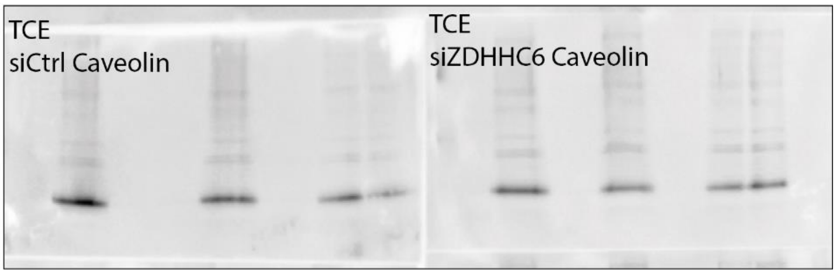

Fig S2b

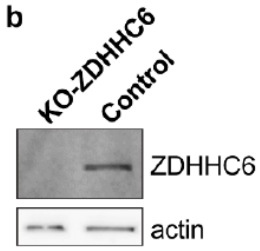

ZDHHC6

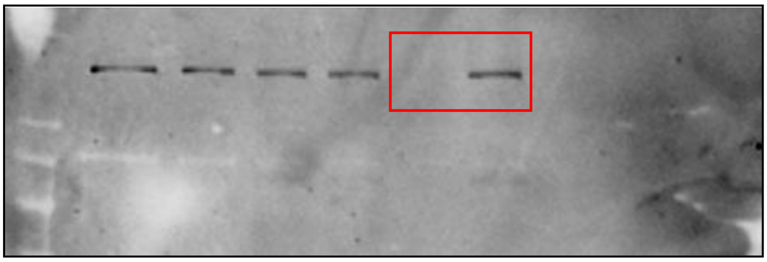

Actin

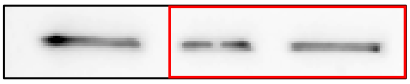

Fig S2c

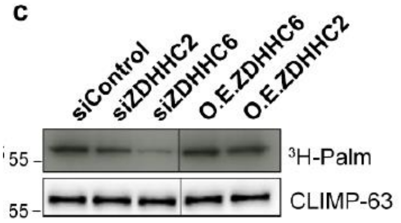

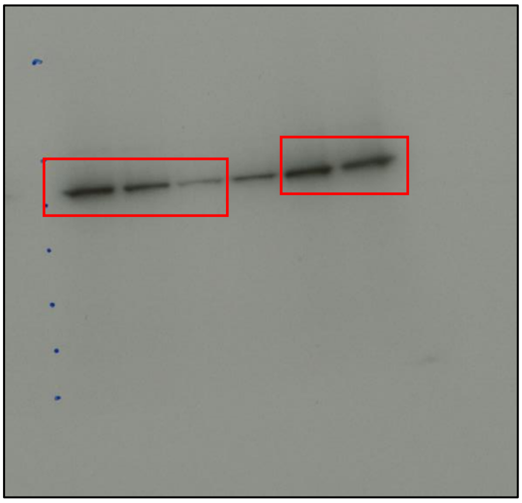

Autoradiography

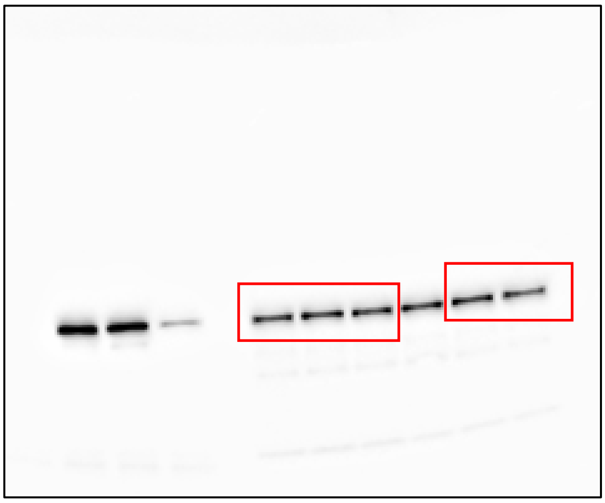

WB anti- climp63

Fig S2e

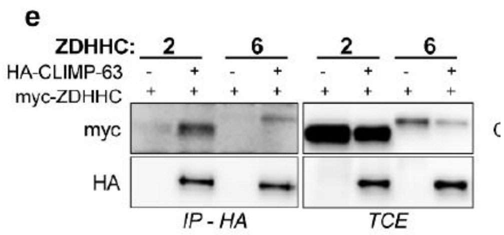

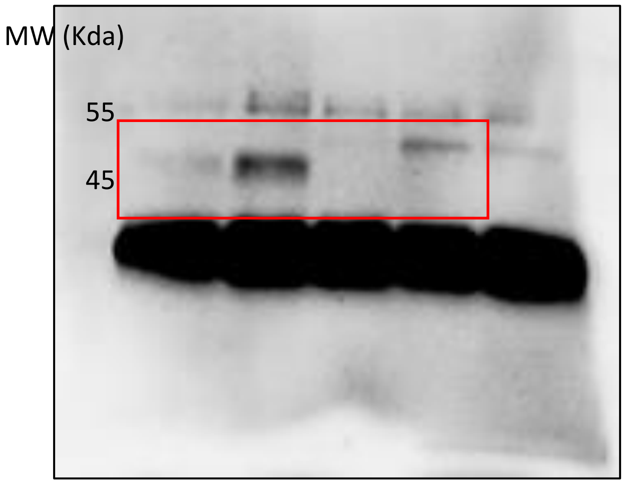

WB anti- myc (IP)

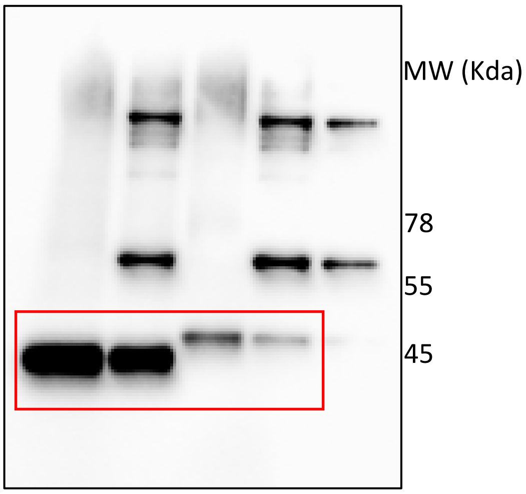

WB anti- myc (TCE)

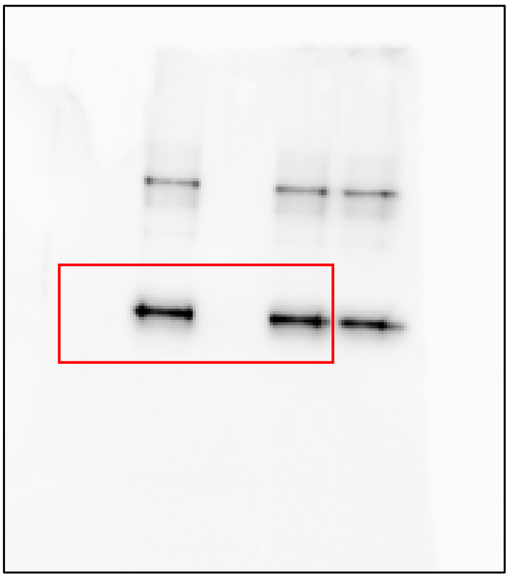

WB anti- HA (IP)

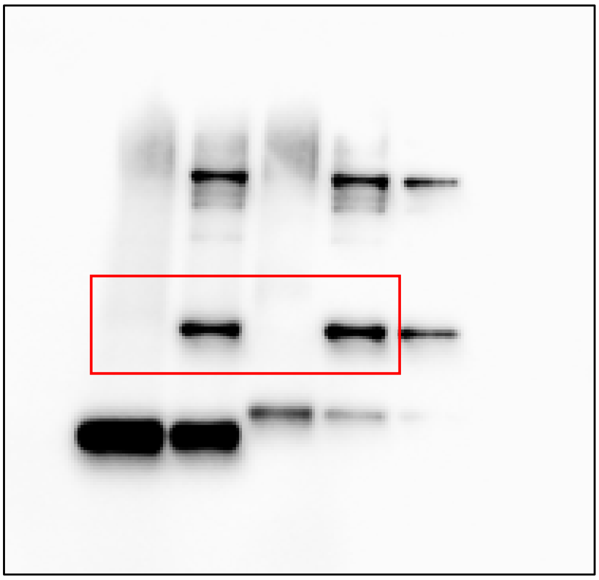

WB anti- HA (TCE)

Fig S2f

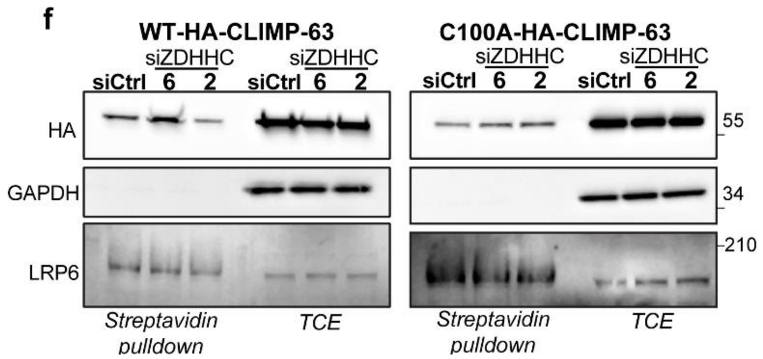

Figure S2f

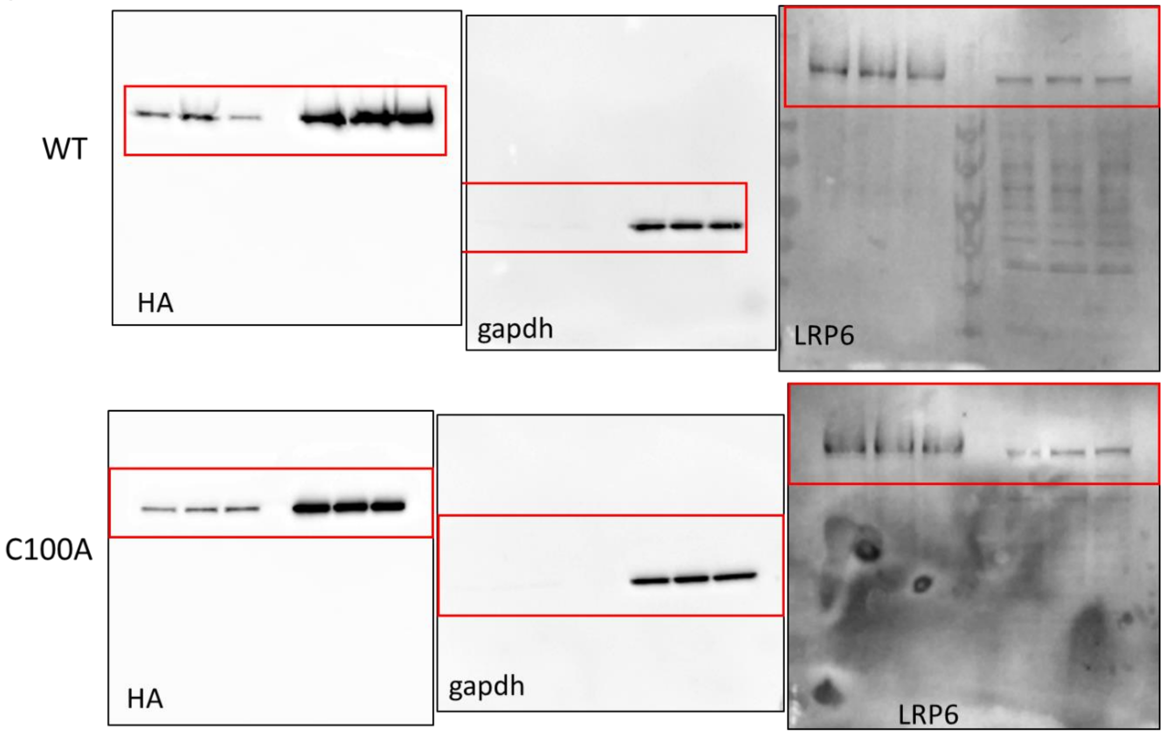

Fig S2h and Fig 6h

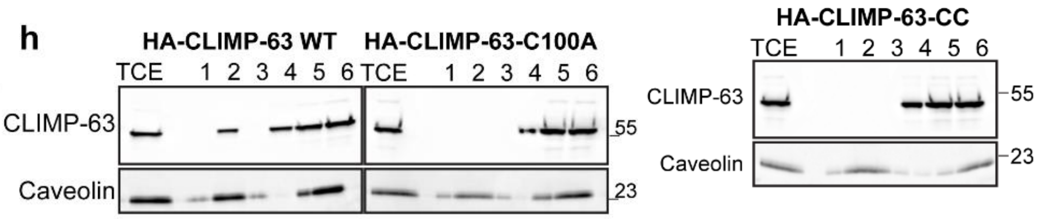

HA

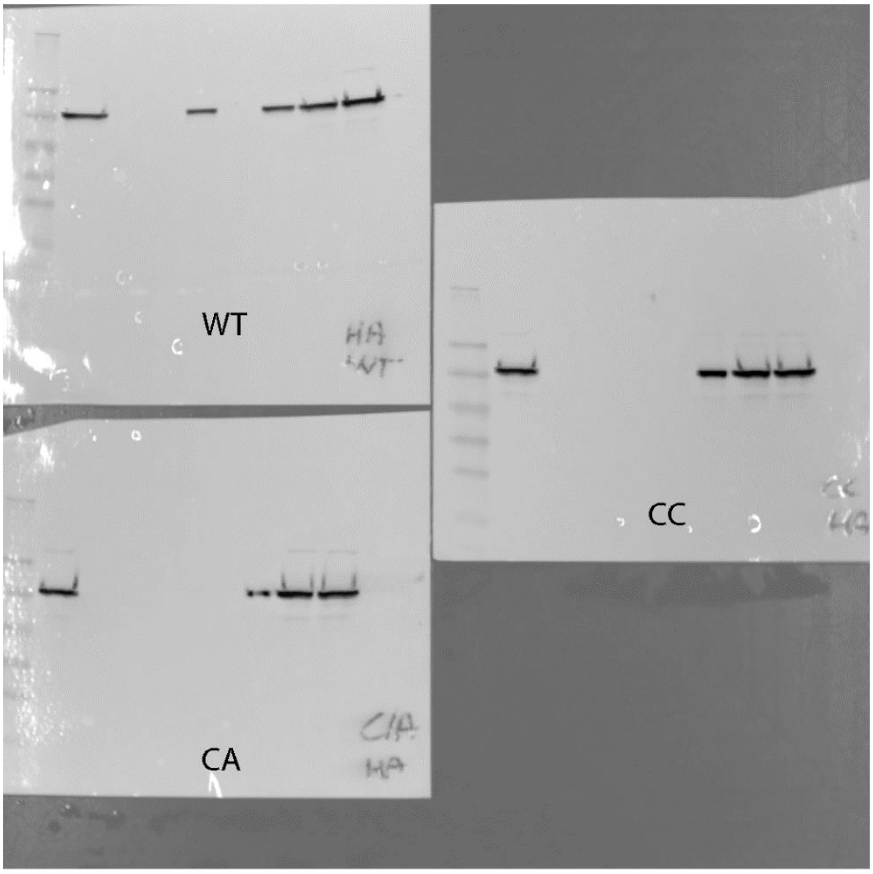

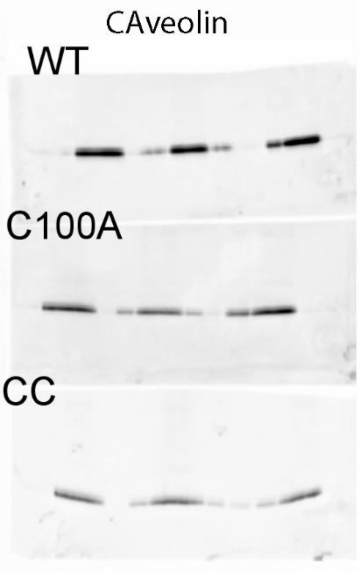

Fig 3e

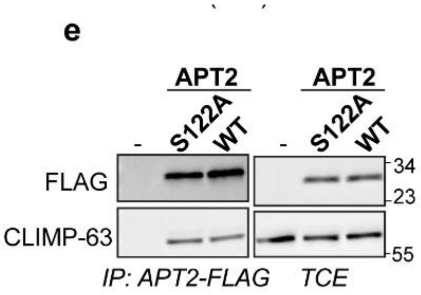

Figure 3e

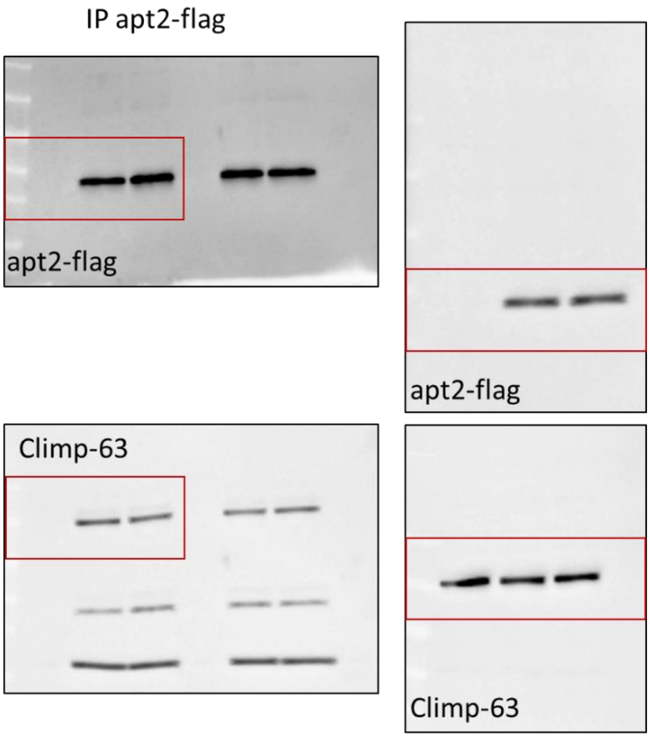

Fig S3a and S3b Patrick

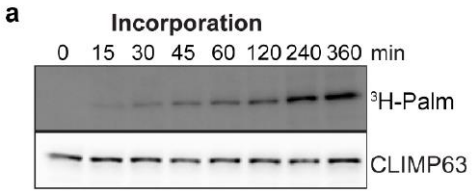

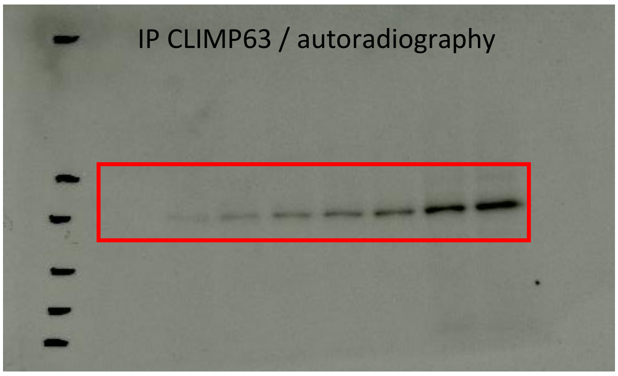

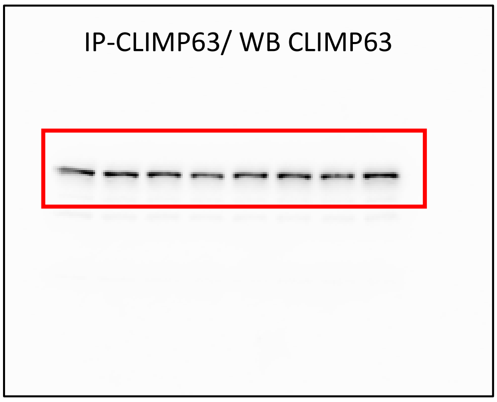

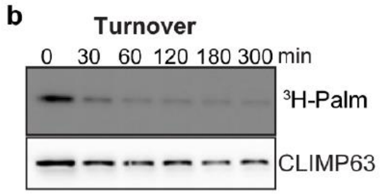

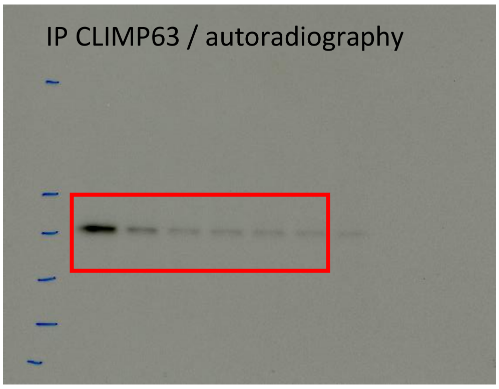

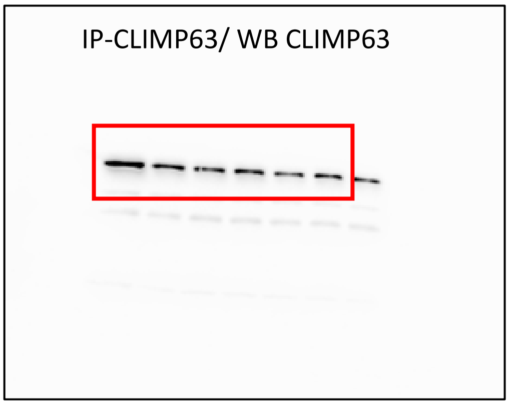

Fig S3c and S3d

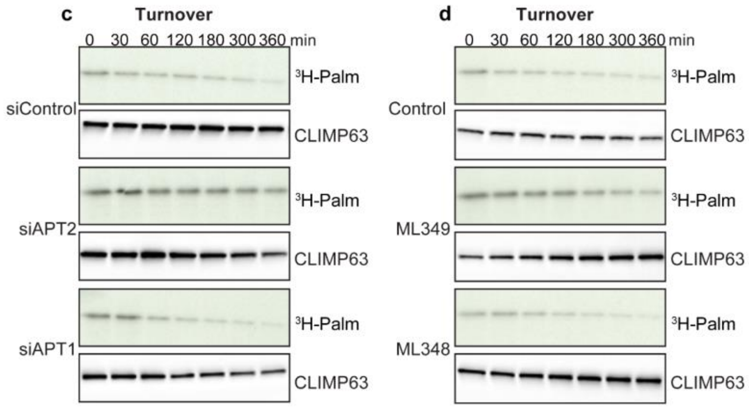

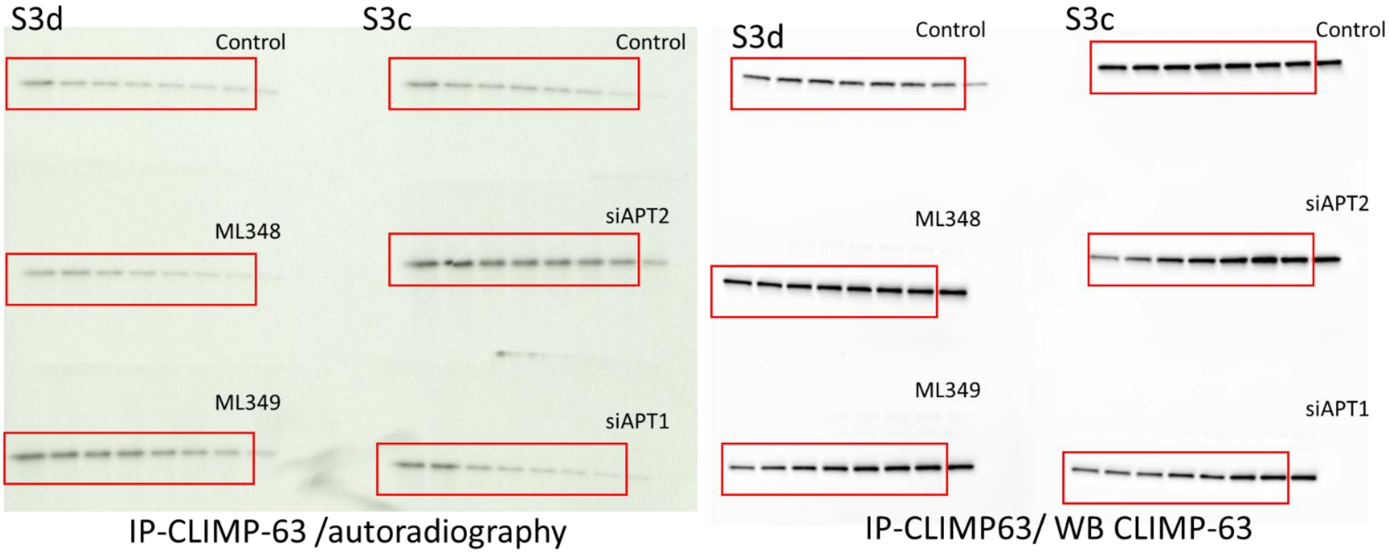

Fig 4b Patrick:

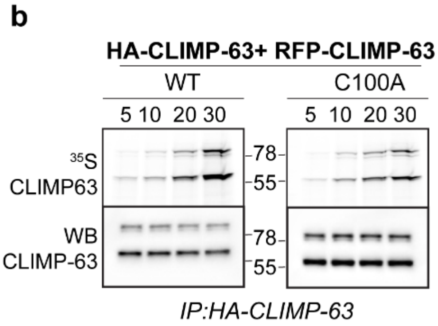

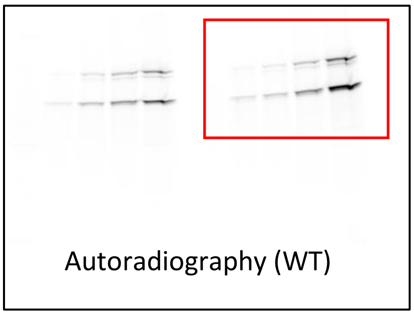
Autoradiography (WT)

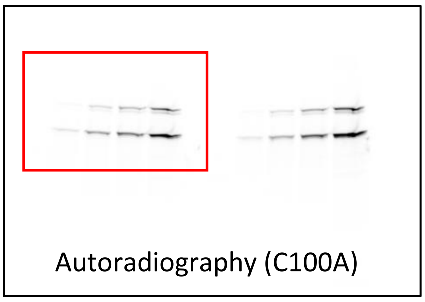
Autoradiography (C100A)

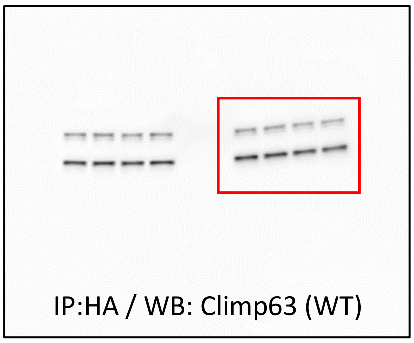
IP:HA / WB: Climp63 (WT)

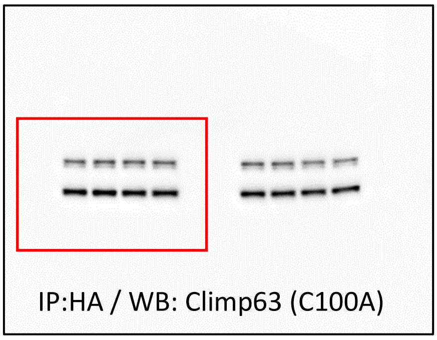
IP:HA / WB: Climp63 (C100A)

Fig 4c

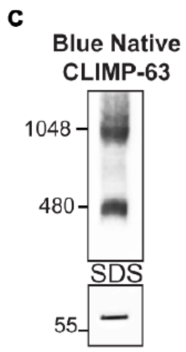

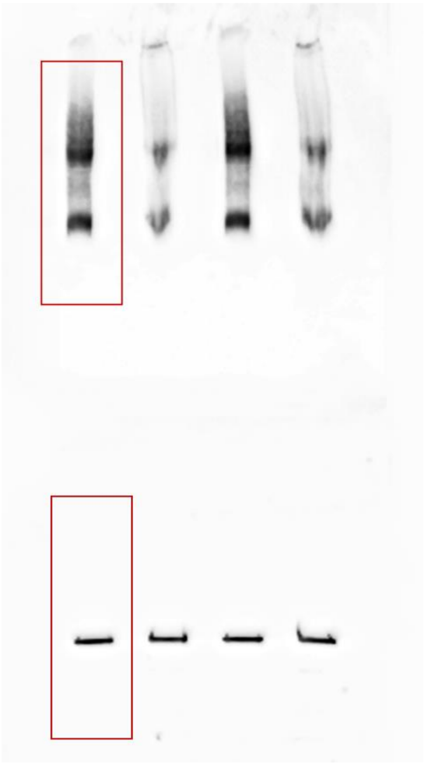

Fig 4d

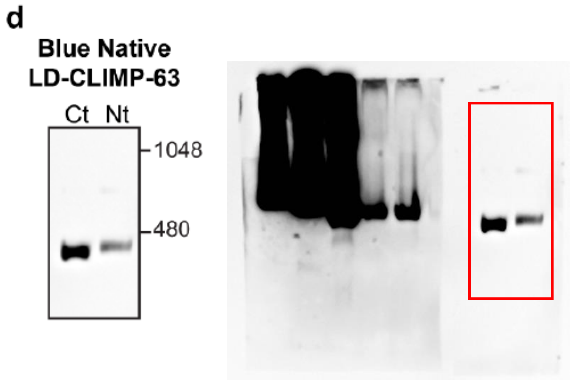

Fig 4e

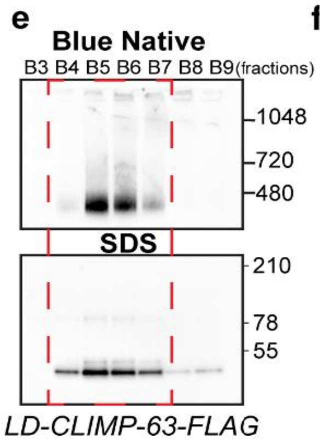
e
f
Blue Native
B3 B4 B5 B6 B7 B8 B9(fractions)
1048
720
480
SDS
210
78
55
LD-CLIMP-63-FLAG

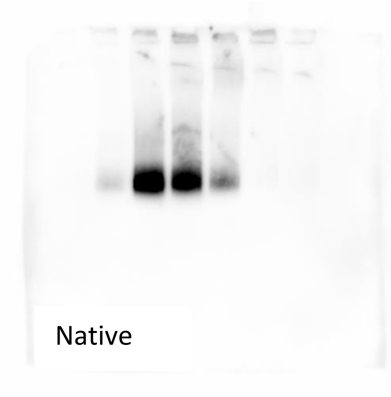
Native

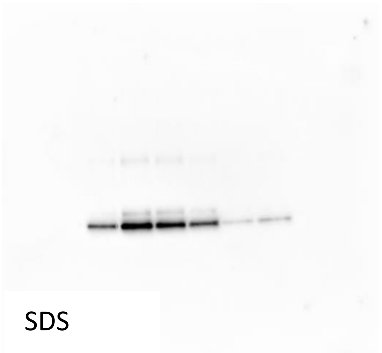
SDS

Fig S4b

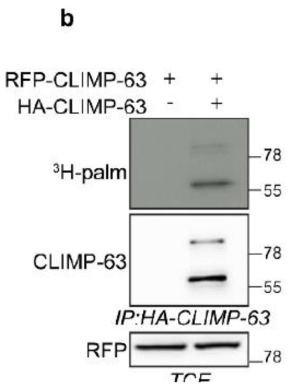

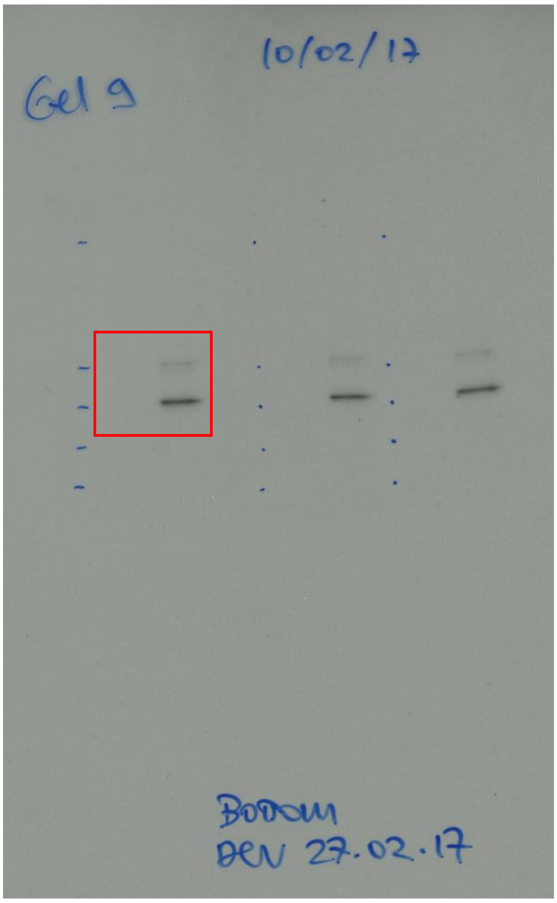

Autoradiography

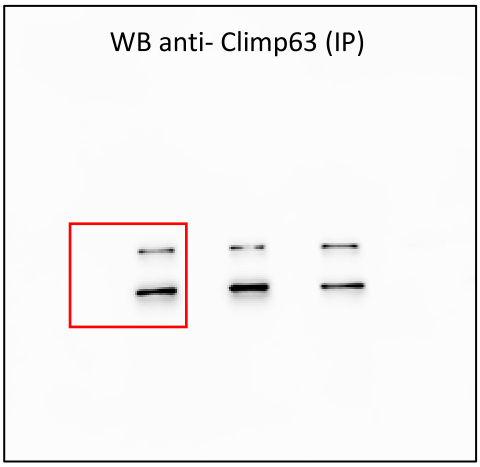

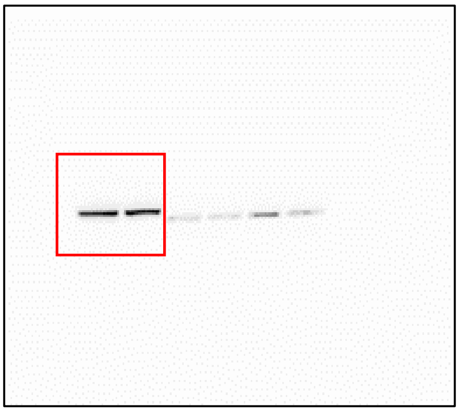

Fig 5g

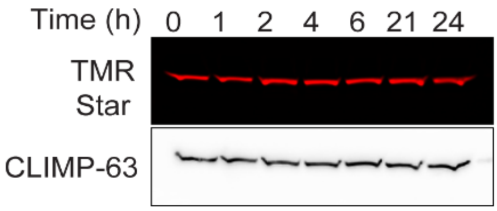

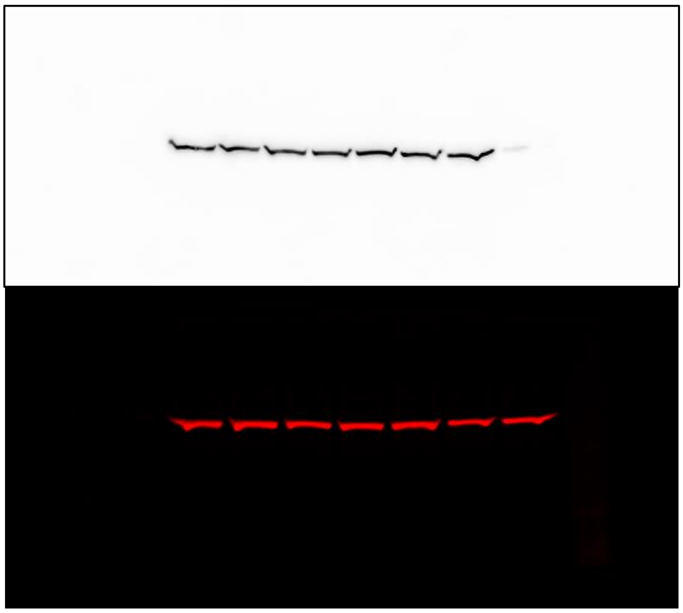

Fig 6g

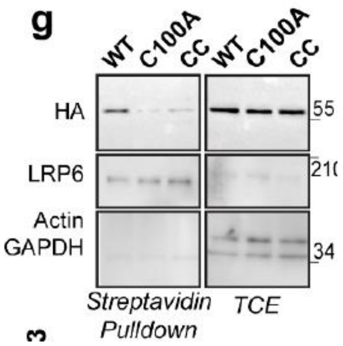

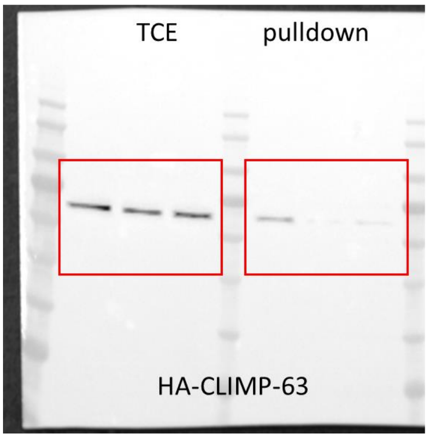

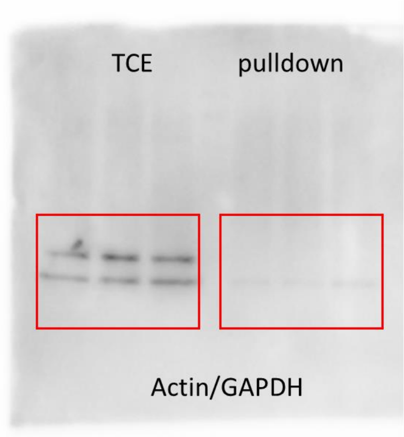

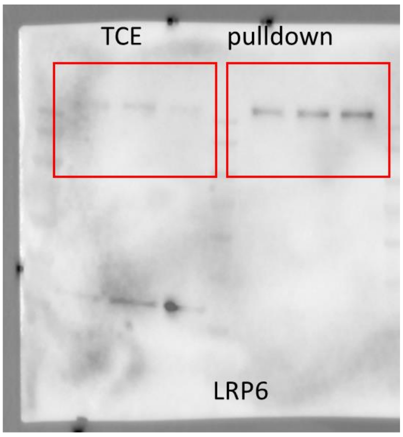

Fig 6h (together with S2h see above)

Fig S6f

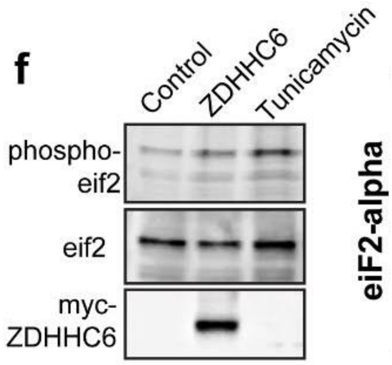

Figure S6f

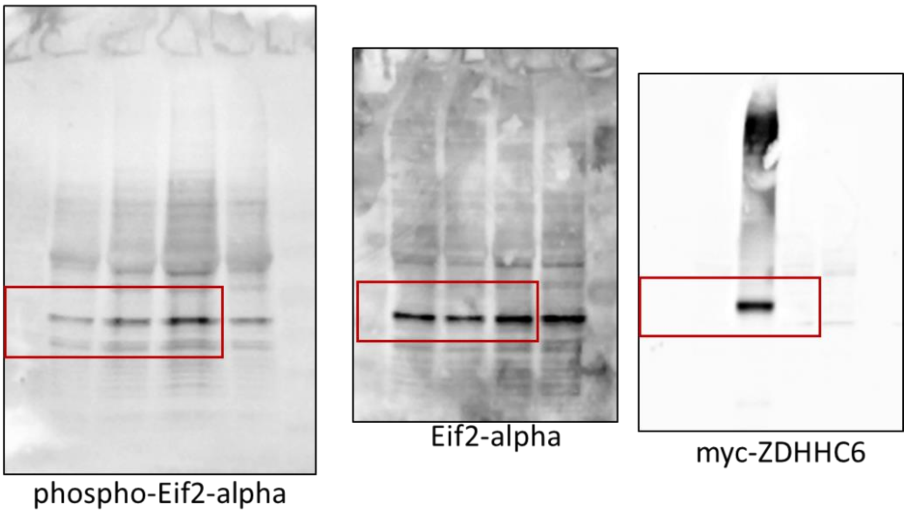

Fig S6i

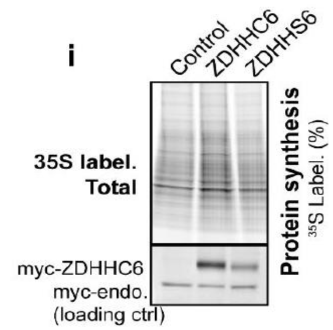

Figure S6i

autoradiography

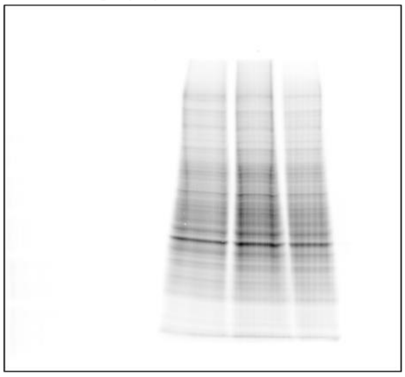

myc-ZDHHC6

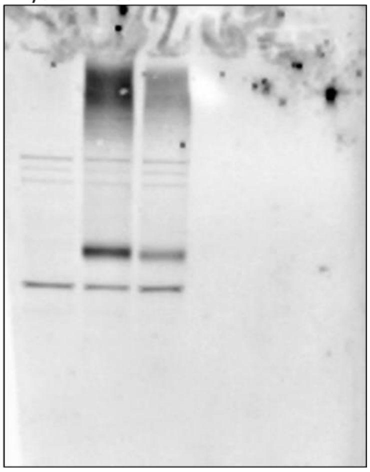

Fig S6j

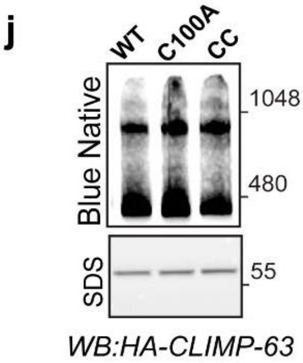

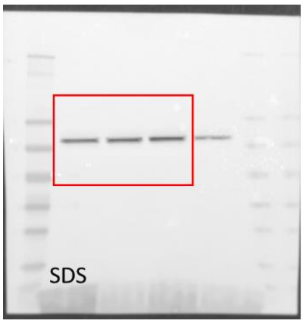

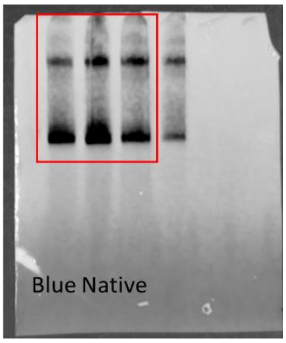

Supplement: Supplementary file 4 — Source Data [file 41467_2023_35921_MOESM4_ESM.zip › Western_Blots.pdf]
